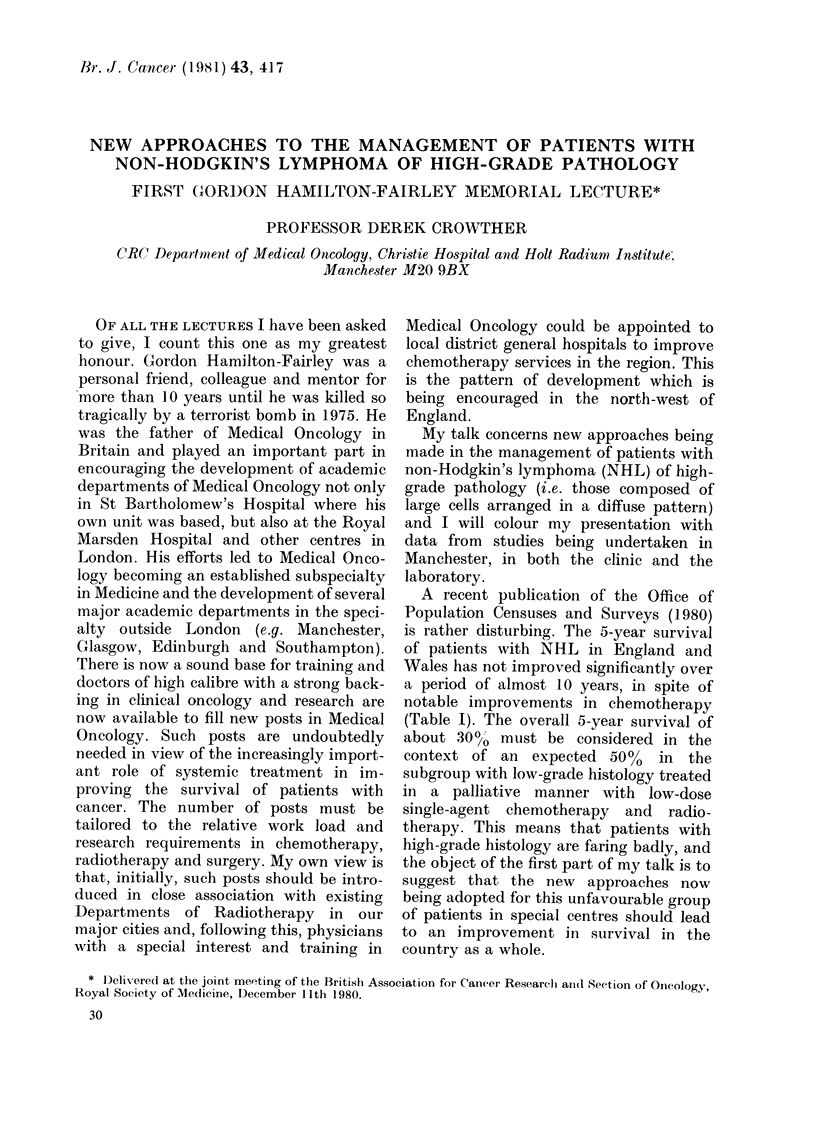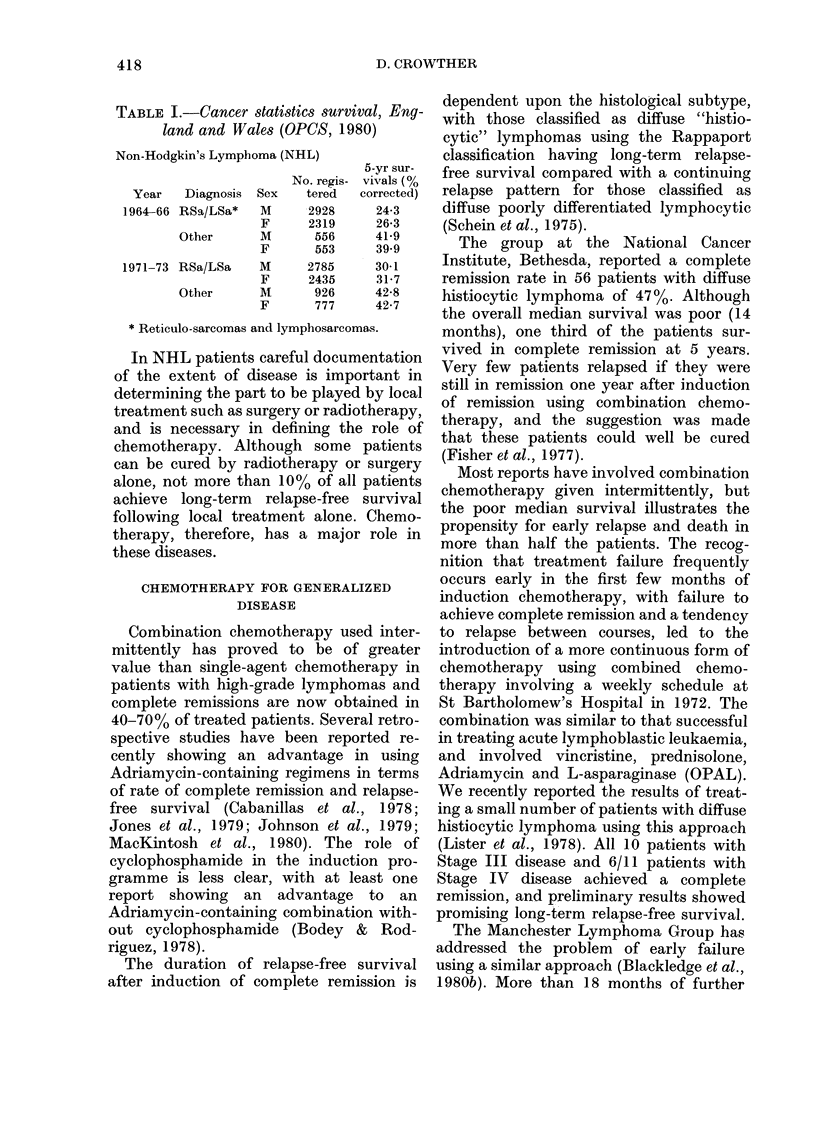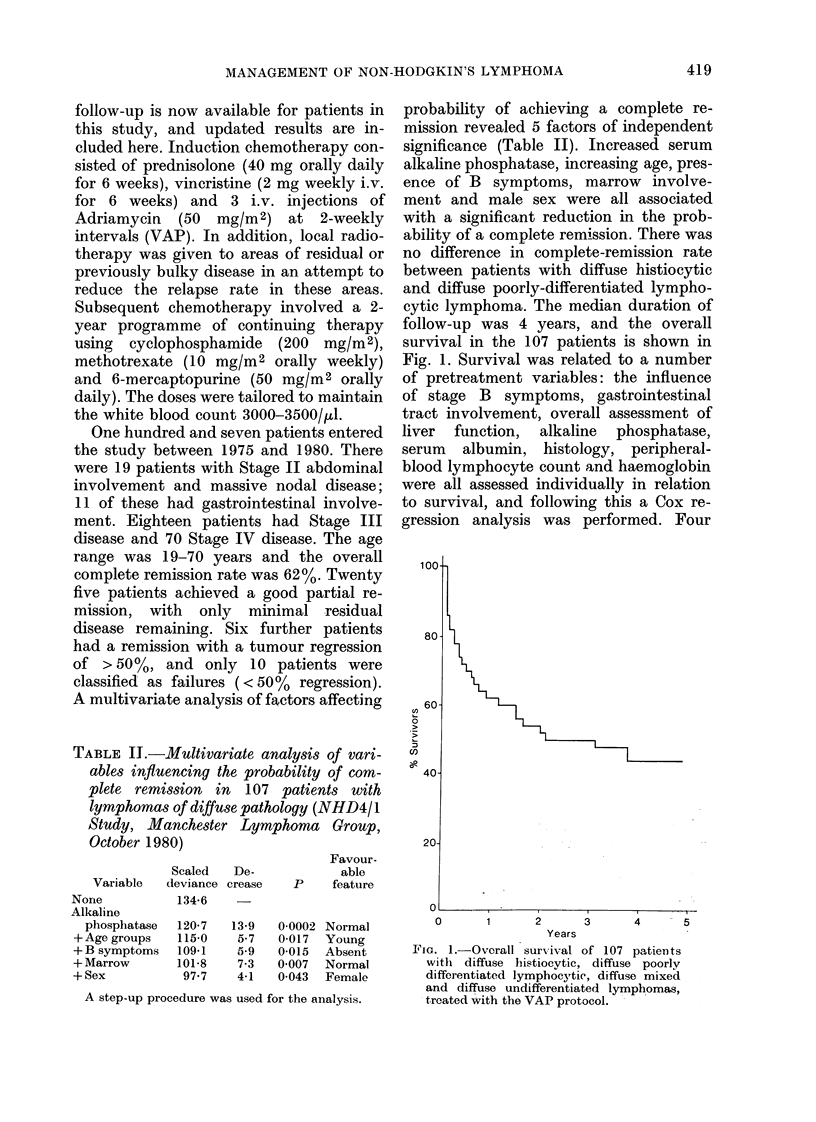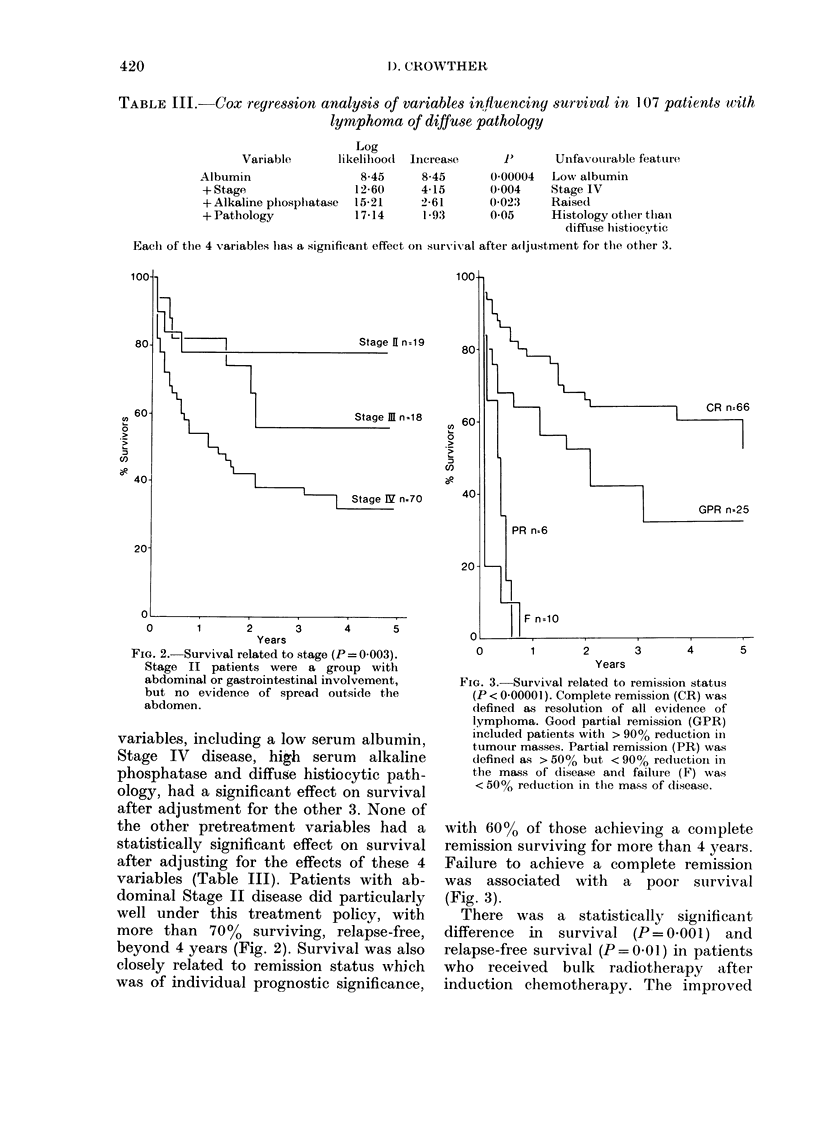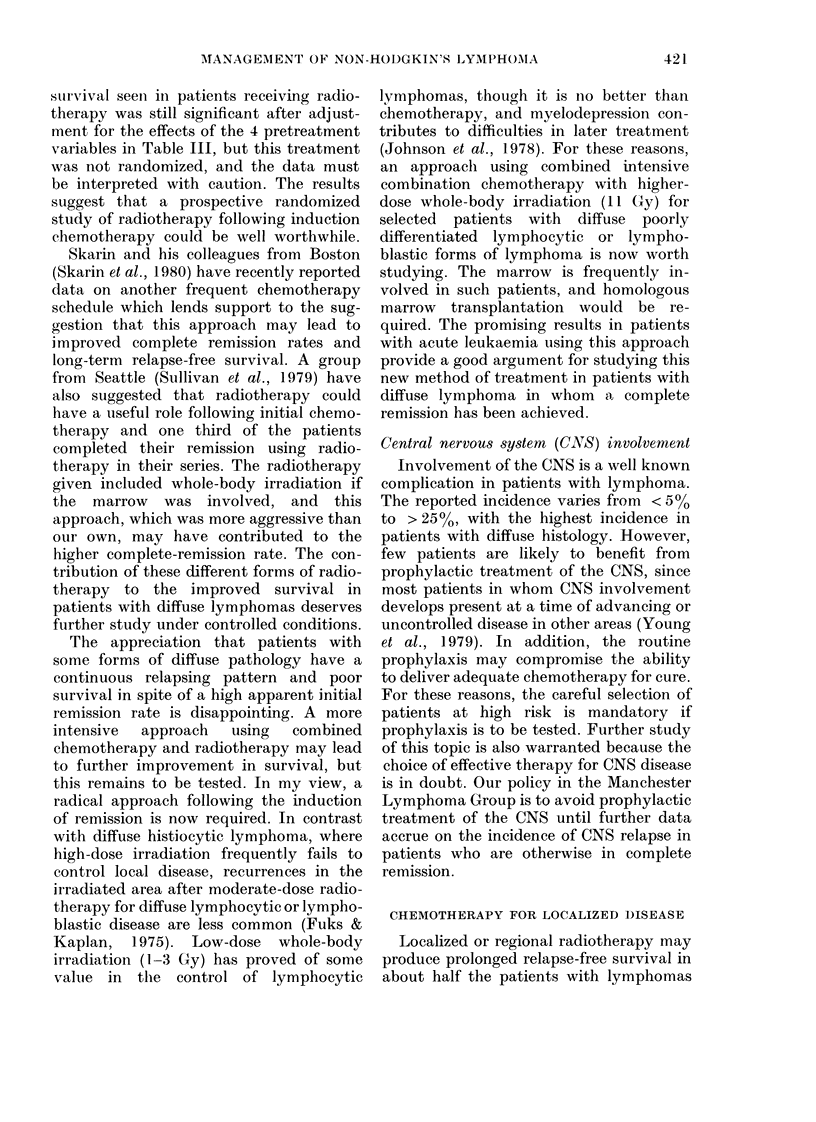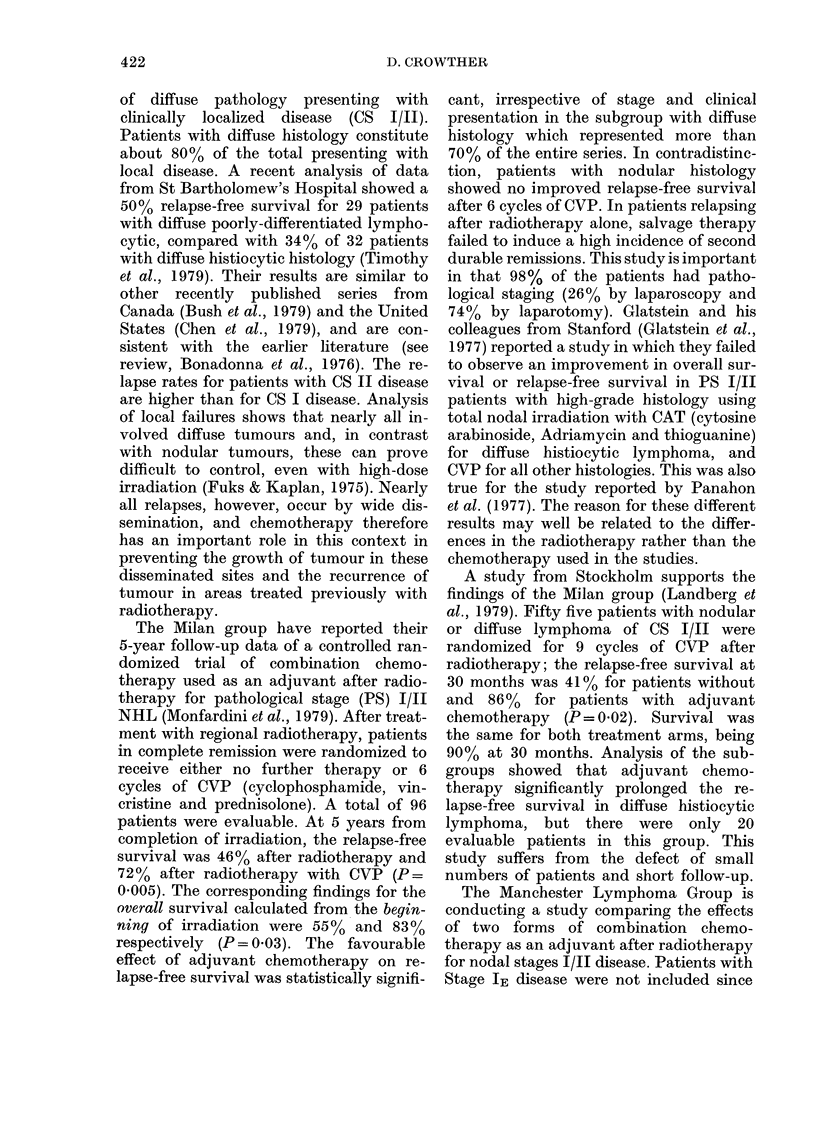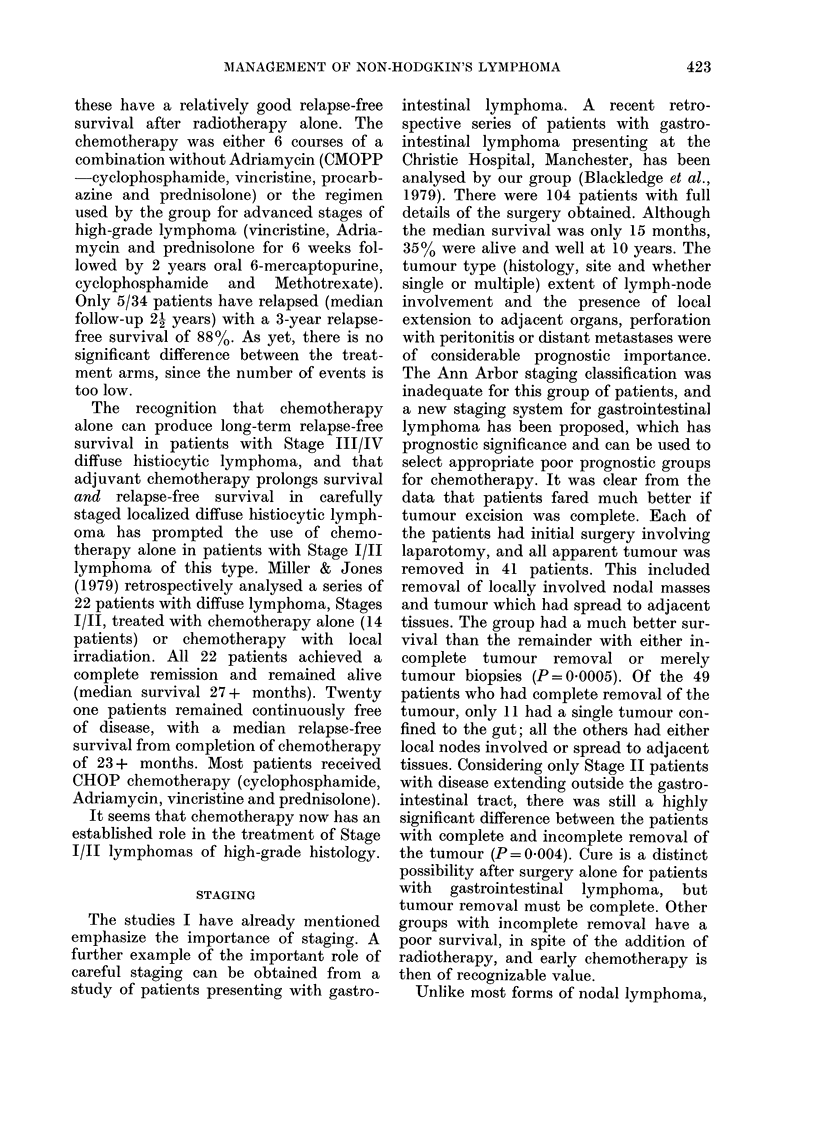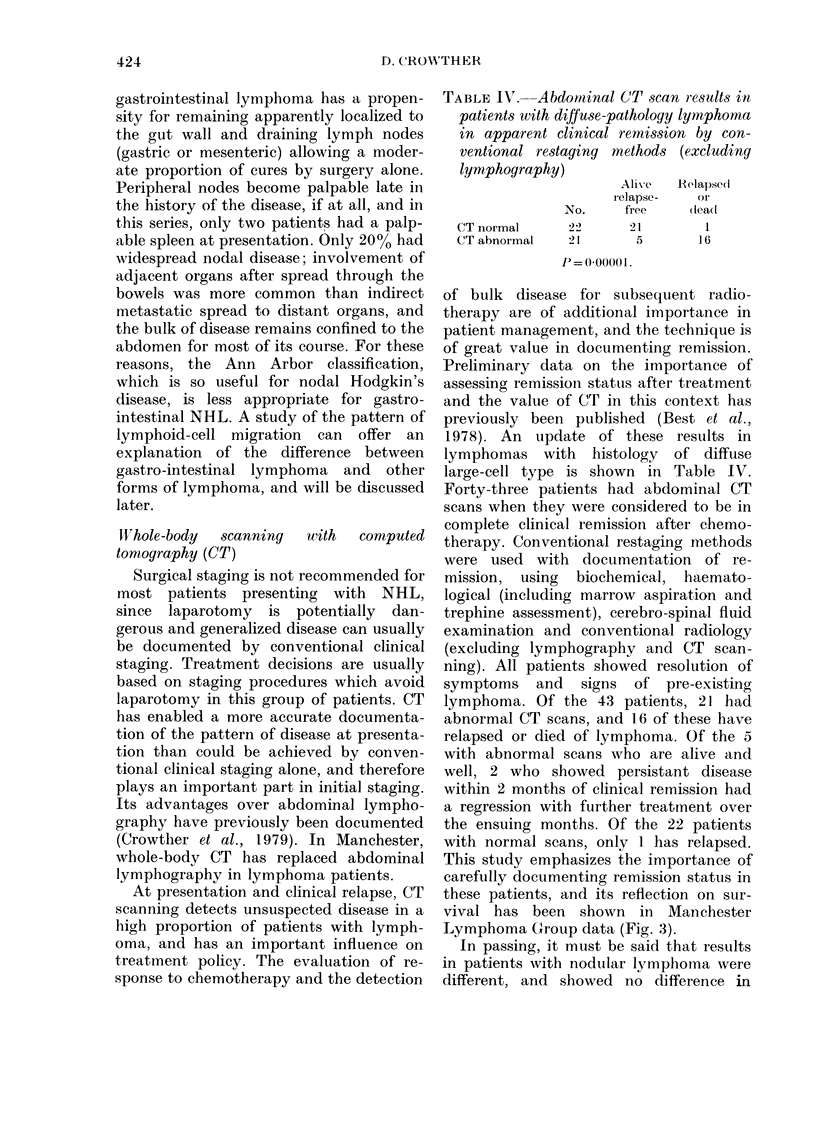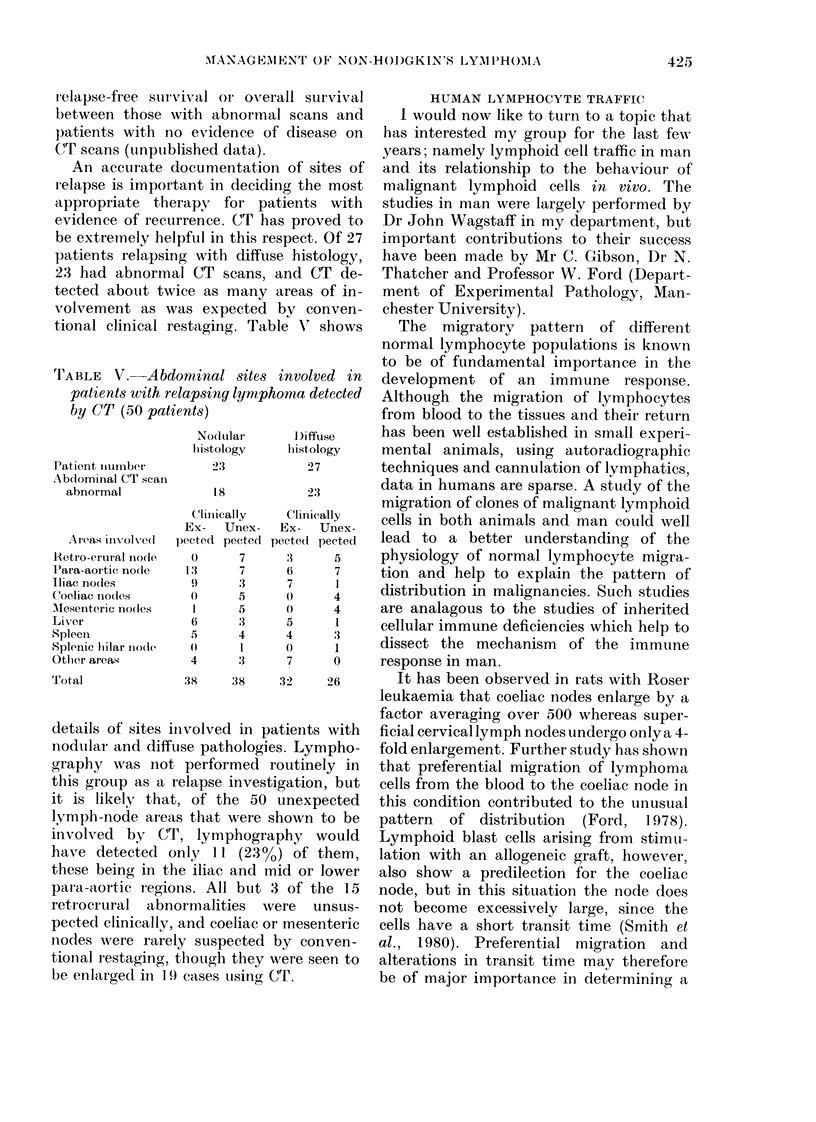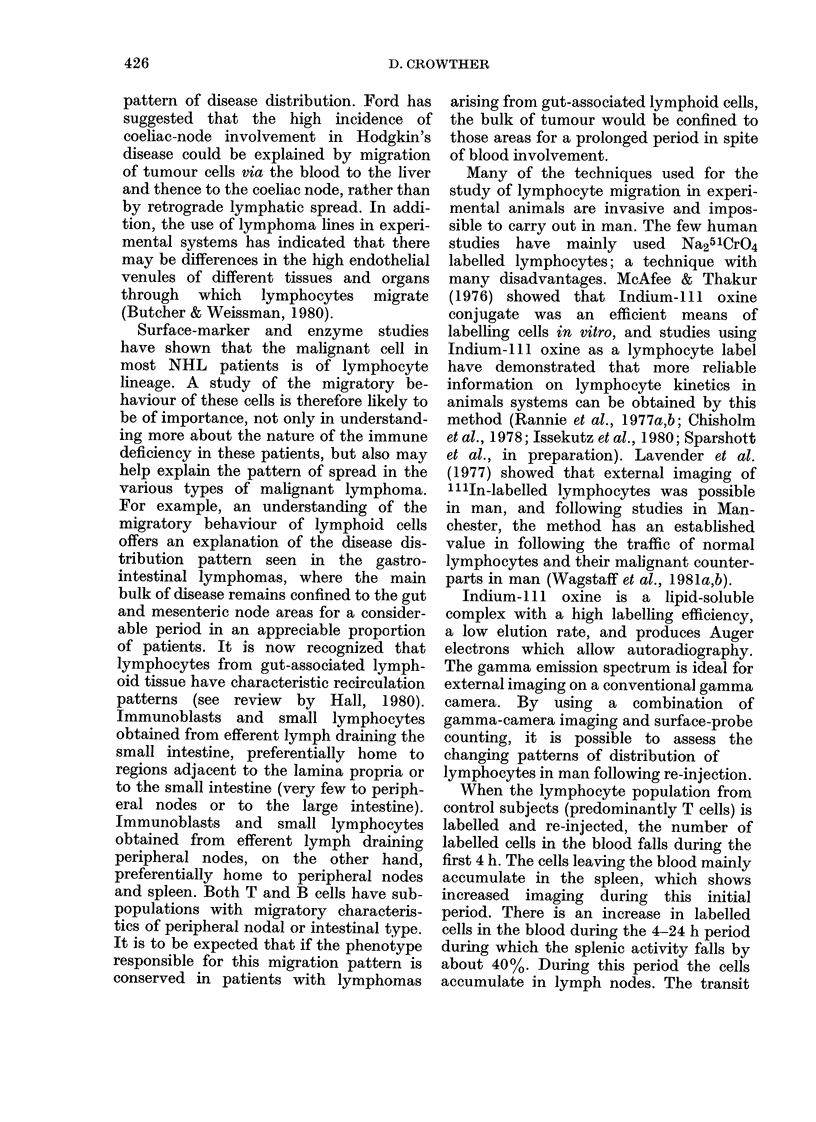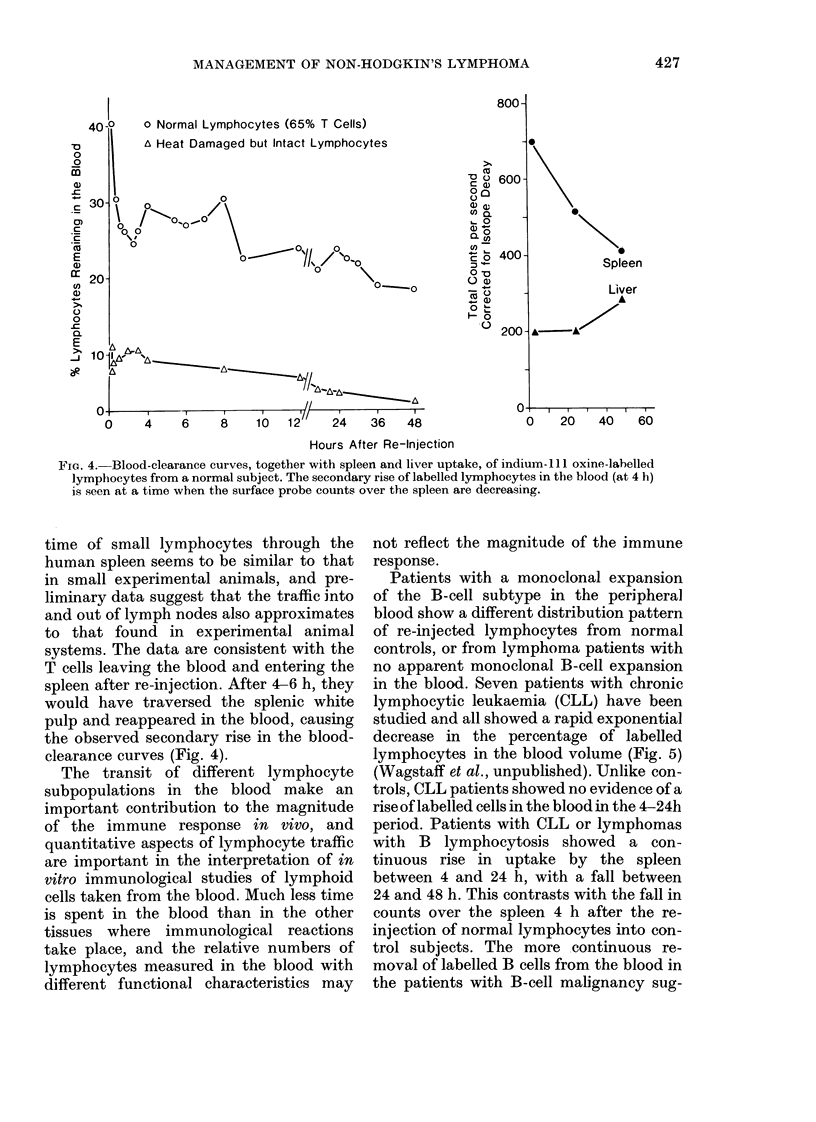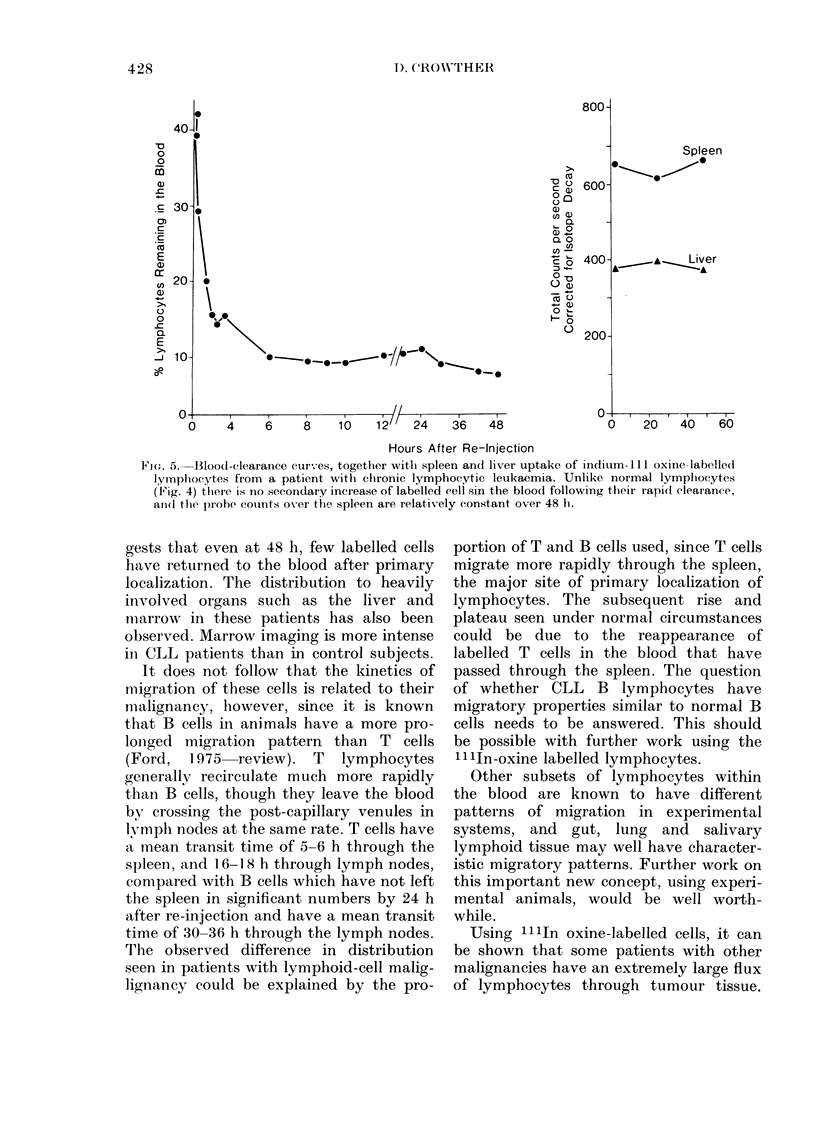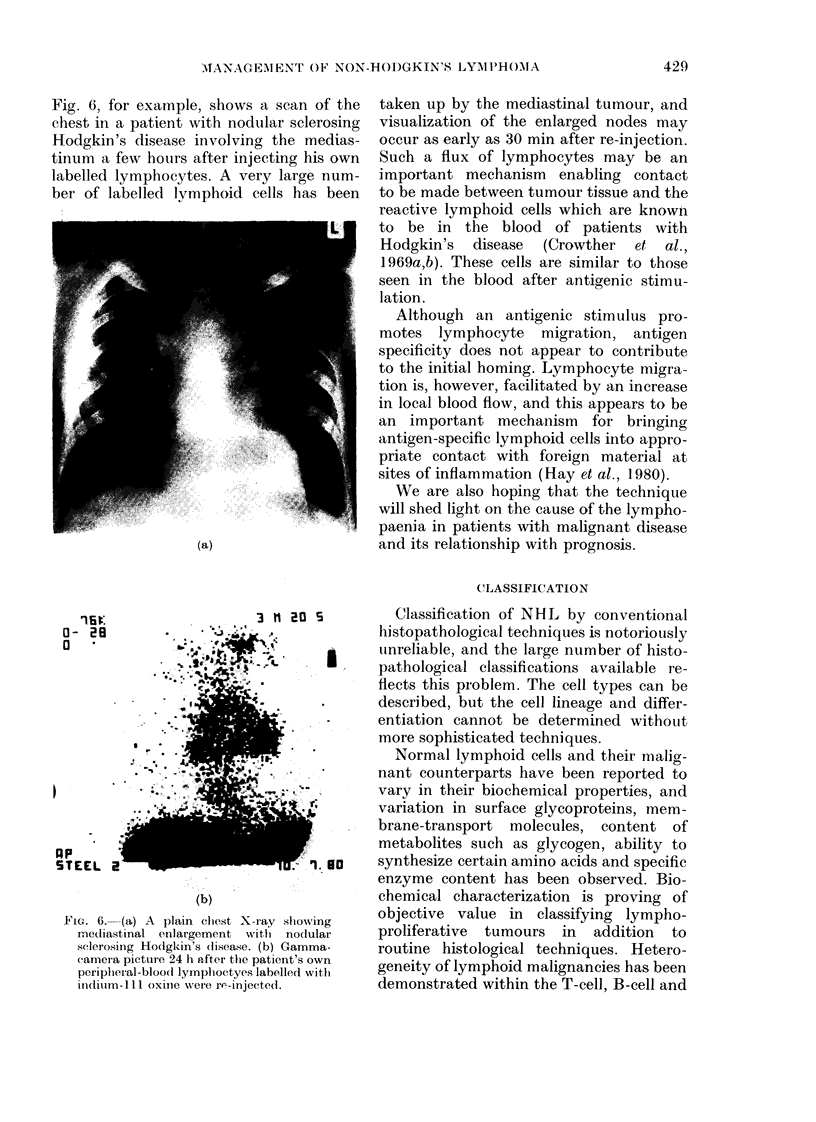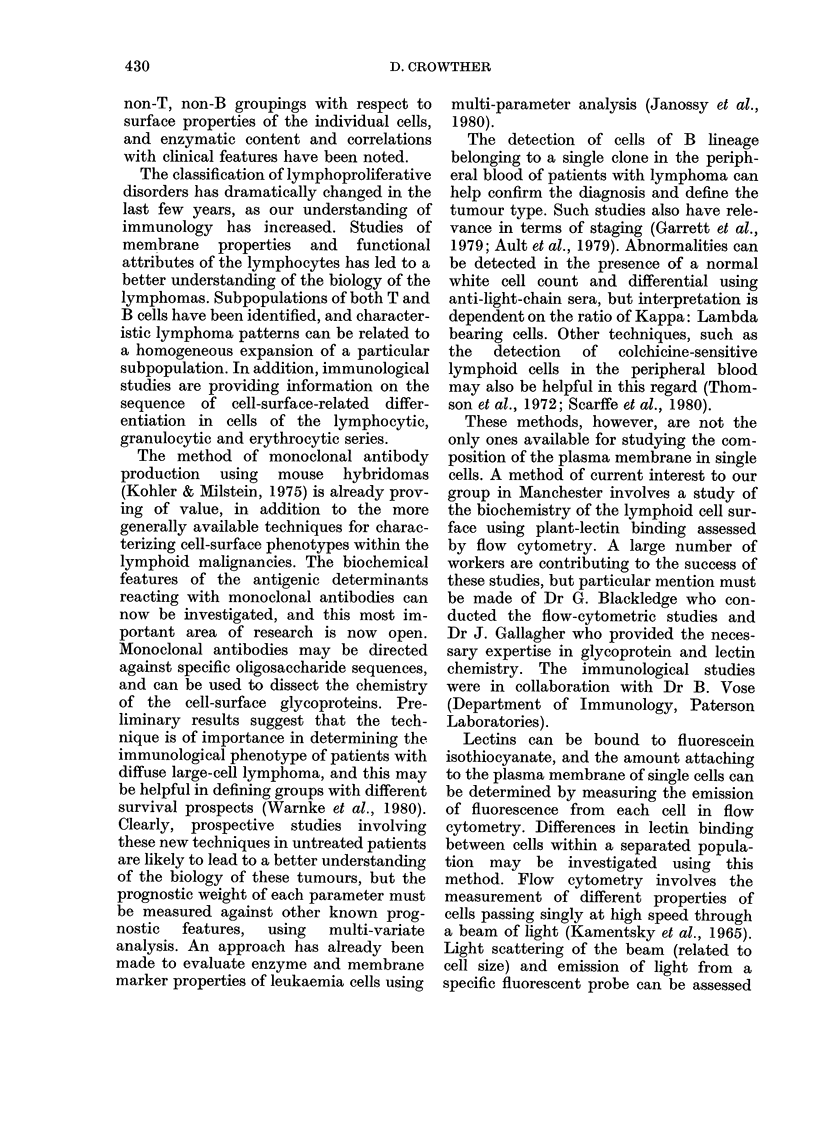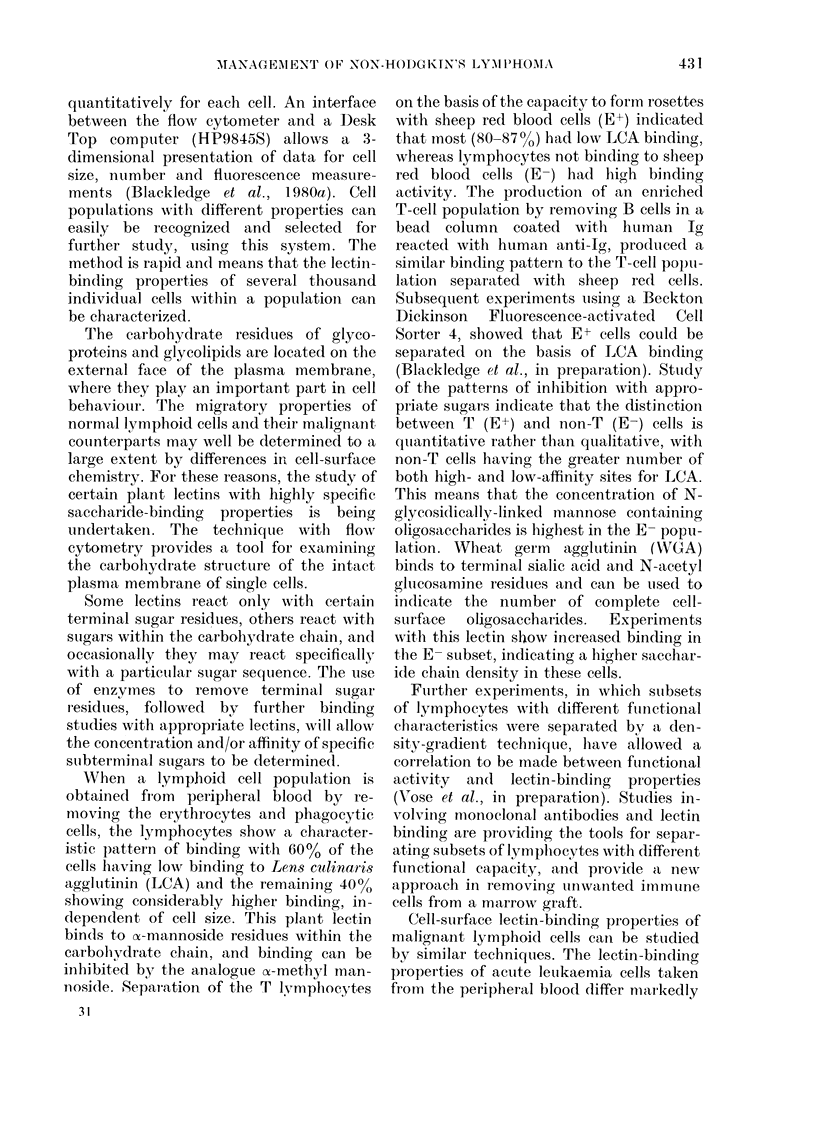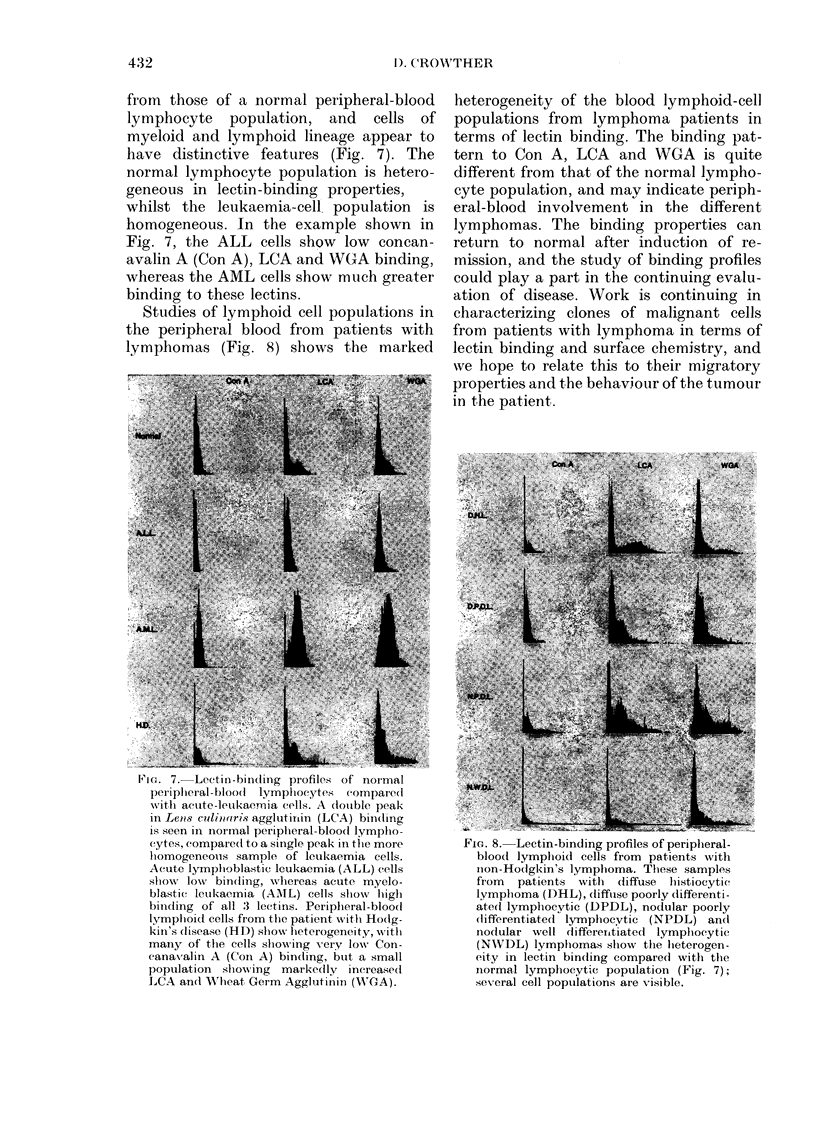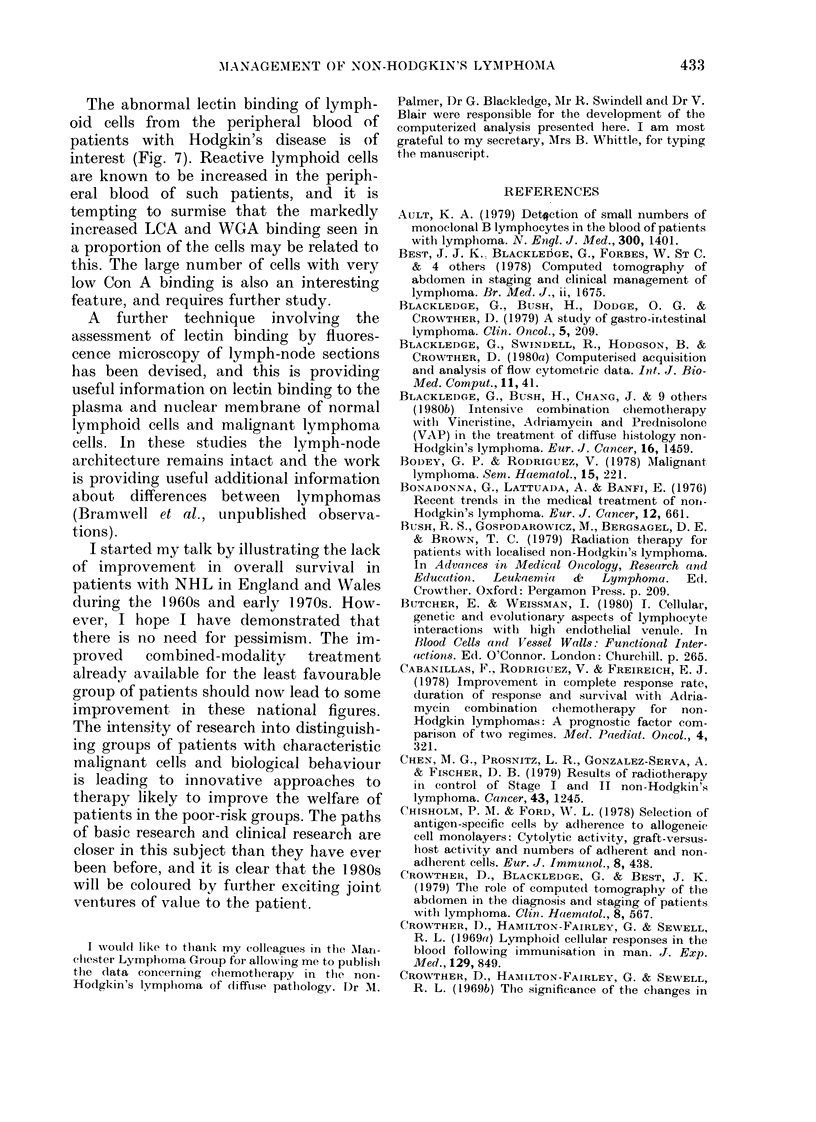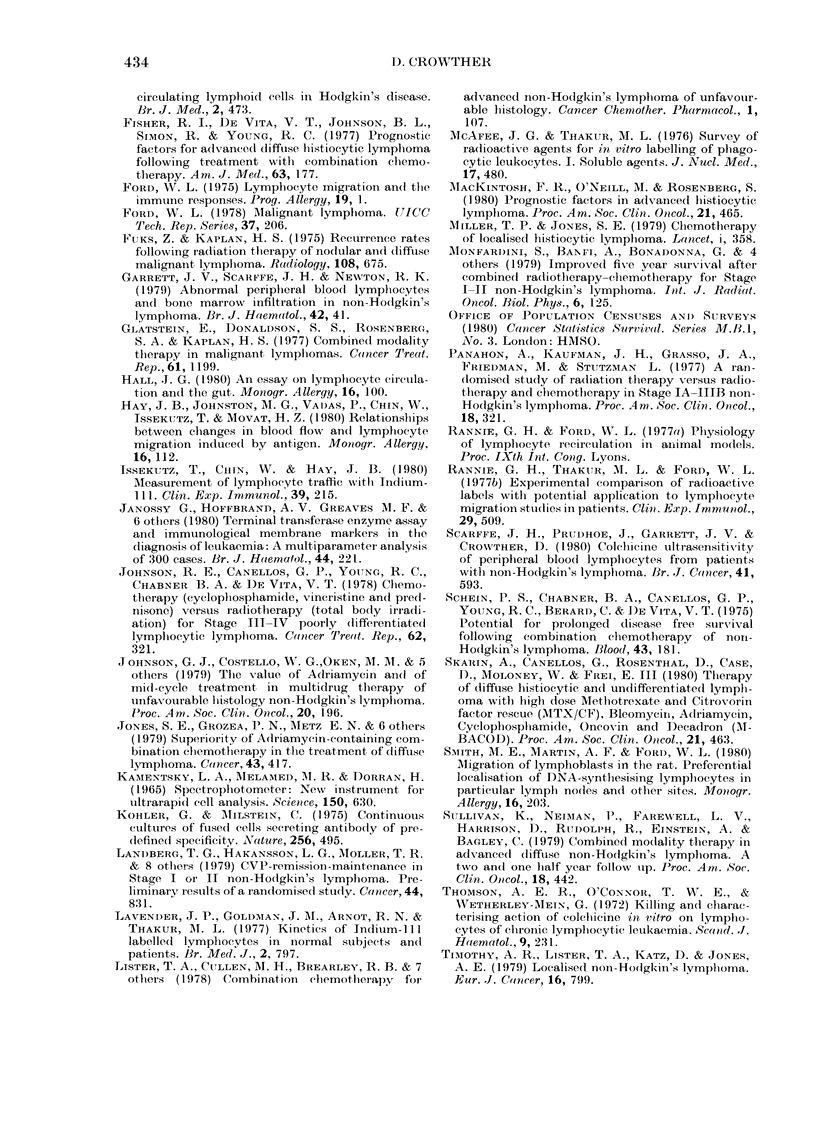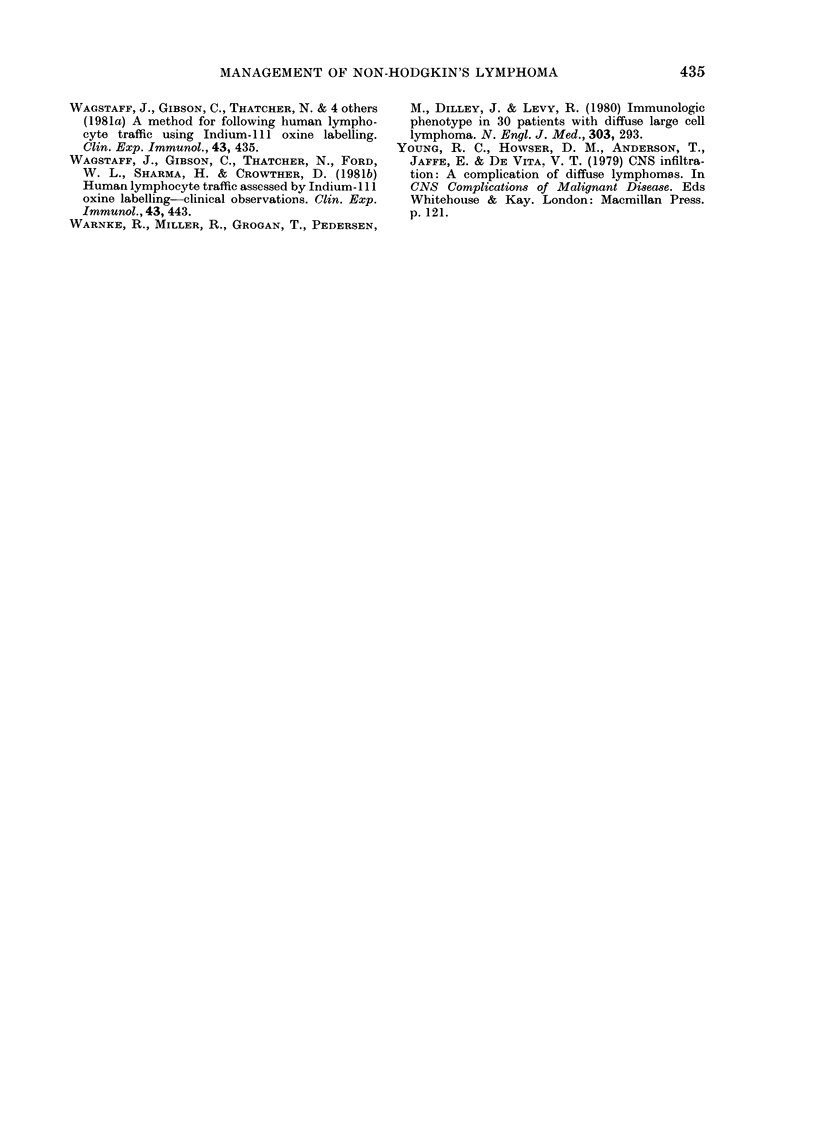# New approaches to the management of patients with non-Hodgkin's lymphoma of high-grade pathology. First Gordon Hamilton-Fairley memorial lecture.

**DOI:** 10.1038/bjc.1981.63

**Published:** 1981-04

**Authors:** D. Crowther

## Abstract

**Images:**


*Br. J. Cancer* (1981) **43**, 417

### NEW APPROACHES TO THE MANAGEMENT OF PATIENTS WITH NON-HODGKIN'S LYMPHOMA OF HIGH-GRADE PATHOLOGY

### FIRST GORDON HAMILTON-FAIRLEY MEMORIAL LECTURE*

PROFESSOR DEREK CROWTHER

*CRC Department of Medical Oncology, Christie Hospital and Holt Radium Institute, Manchester M20 9BX*

OF ALL THE LECTURES I have been asked to give, I count this one as my greatest honour. Gordon Hamilton-Fairley was a personal friend, colleague and mentor for more than 10 years until he was killed so tragically by a terrorist bomb in 1975. He was the father of Medical Oncology in Britain and played an important part in encouraging the development of academic departments of Medical Oncology not only in St Bartholomew's Hospital where his own unit was based, but also at the Royal Marsden Hospital and other centres in London. His efforts led to Medical Oncology becoming an established subspecialty in Medicine and the development of several major academic departments in the specialty outside London (*e.g.* Manchester, Glasgow, Edinburgh and Southampton). There is now a sound base for training and doctors of high calibre with a strong backing in clinical oncology and research are now available to fill new posts in Medical Oncology. Such posts are undoubtedly needed in view of the increasingly important role of systemic treatment in improving the survival of patients with cancer. The number of posts must be tailored to the relative work load and research requirements in chemotherapy, radiotherapy and surgery. My own view is that, initially, such posts should be introduced in close association with existing Departments of Radiotherapy in our major cities and, following this, physicians with a special interest and training in Medical Oncology could be appointed to local district general hospitals to improve chemotherapy services in the region. This is the pattern of development which is being encouraged in the north-west of England.

My talk concerns new approaches being made in the management of patients with non-Hodgkin's lymphoma (NHL) of high-grade pathology (*i.e.* those composed of large cells arranged in a diffuse pattern) and I will colour my presentation with data from studies being undertaken in Manchester, in both the clinic and the laboratory.

A recent publication of the Office of Population Censuses and Surveys (1980) is rather disturbing. The 5-year survival of patients with NHL in England and Wales has not improved significantly over a period of almost 10 years, in spite of notable improvements in chemotherapy (Table I). The overall 5-year survival of about 30% must be considered in the context of an expected 50% in the subgroup with low-grade histology treated in a palliative manner with low-dose single-agent chemotherapy and radiotherapy. This means that patients with high-grade histology are faring badly, and the object of the first part of my talk is to suggest that the new approaches now being adopted for this unfavourable group of patients in special centres should lead to an improvement in survival in the country as a whole.

* Delivered at the joint meeting of the British Association for Cancer Research and Section of Oncology, Royal Society of Medicine, December 11th 1980.

TABLE I.—*Cancer statistics survival, England and Wales* (*OPCS*, 1980)

Non-Hodgkin's Lymphoma (NHL)

| Year | Diagnosis | Sex | No. registered | 5-yr survivals (% corrected) |
|---|---|---|---|---|
| 1964–66 | RSa/LSa* | M | 2928 | 24·3 |
| | | F | 2319 | 26·3 |
| | Other | M | 556 | 41·9 |
| | | F | 553 | 39·9 |
| 1971–73 | RSa/LSa | M | 2785 | 30·1 |
| | | F | 2435 | 31·7 |
| | Other | M | 926 | 42·8 |
| | | F | 777 | 42·7 |

* Reticulo-sarcomas and lymphosarcomas.

In NHL patients careful documentation of the extent of disease is important in determining the part to be played by local treatment such as surgery or radiotherapy, and is necessary in defining the role of chemotherapy. Although some patients can be cured by radiotherapy or surgery alone, not more than 10% of all patients achieve long-term relapse-free survival following local treatment alone. Chemotherapy, therefore, has a major role in these diseases.

### CHEMOTHERAPY FOR GENERALIZED DISEASE

Combination chemotherapy used intermittently has proved to be of greater value than single-agent chemotherapy in patients with high-grade lymphomas and complete remissions are now obtained in 40–70% of treated patients. Several retrospective studies have been reported recently showing an advantage in using Adriamycin-containing regimens in terms of rate of complete remission and relapse-free survival (Cabanillas *et al.*, 1978; Jones *et al.*, 1979; Johnson *et al.*, 1979; MacKintosh *et al.*, 1980). The role of cyclophosphamide in the induction programme is less clear, with at least one report showing an advantage to an Adriamycin-containing combination without cyclophosphamide (Bodey & Rodriguez, 1978).

The duration of relapse-free survival after induction of complete remission is dependent upon the histological subtype, with those classified as diffuse "histiocytic" lymphomas using the Rappaport classification having long-term relapse-free survival compared with a continuing relapse pattern for those classified as diffuse poorly differentiated lymphocytic (Schein *et al.*, 1975).

The group at the National Cancer Institute, Bethesda, reported a complete remission rate in 56 patients with diffuse histiocytic lymphoma of 47%. Although the overall median survival was poor (14 months), one third of the patients survived in complete remission at 5 years. Very few patients relapsed if they were still in remission one year after induction of remission using combination chemotherapy, and the suggestion was made that these patients could well be cured (Fisher *et al.*, 1977).

Most reports have involved combination chemotherapy given intermittently, but the poor median survival illustrates the propensity for early relapse and death in more than half the patients. The recognition that treatment failure frequently occurs early in the first few months of induction chemotherapy, with failure to achieve complete remission and a tendency to relapse between courses, led to the introduction of a more continuous form of chemotherapy using combined chemotherapy involving a weekly schedule at St Bartholomew's Hospital in 1972. The combination was similar to that successful in treating acute lymphoblastic leukaemia, and involved vincristine, prednisolone, Adriamycin and L-asparaginase (OPAL). We recently reported the results of treating a small number of patients with diffuse histiocytic lymphoma using this approach (Lister *et al.*, 1978). All 10 patients with Stage III disease and 6/11 patients with Stage IV disease achieved a complete remission, and preliminary results showed promising long-term relapse-free survival.

The Manchester Lymphoma Group has addressed the problem of early failure using a similar approach (Blackledge *et al.*, 1980*b*). More than 18 months of further follow-up is now available for patients in this study, and updated results are included here. Induction chemotherapy consisted of prednisolone (40 mg orally daily for 6 weeks), vincristine (2 mg weekly i.v. for 6 weeks) and 3 i.v. injections of Adriamycin (50 mg/m$^2$) at 2-weekly intervals (VAP). In addition, local radiotherapy was given to areas of residual or previously bulky disease in an attempt to reduce the relapse rate in these areas. Subsequent chemotherapy involved a 2-year programme of continuing therapy using cyclophosphamide (200 mg/m$^2$), methotrexate (10 mg/m$^2$ orally weekly) and 6-mercaptopurine (50 mg/m$^2$ orally daily). The doses were tailored to maintain the white blood count 3000–3500/$\mu$l.

One hundred and seven patients entered the study between 1975 and 1980. There were 19 patients with Stage II abdominal involvement and massive nodal disease; 11 of these had gastrointestinal involvement. Eighteen patients had Stage III disease and 70 Stage IV disease. The age range was 19–70 years and the overall complete remission rate was 62%. Twenty five patients achieved a good partial remission, with only minimal residual disease remaining. Six further patients had a remission with a tumour regression of >50%, and only 10 patients were classified as failures (<50% regression). A multivariate analysis of factors affecting probability of achieving a complete remission revealed 5 factors of independent significance (Table II). Increased serum alkaline phosphatase, increasing age, presence of B symptoms, marrow involvement and male sex were all associated with a significant reduction in the probability of a complete remission. There was no difference in complete-remission rate between patients with diffuse histiocytic and diffuse poorly-differentiated lymphocytic lymphoma. The median duration of follow-up was 4 years, and the overall survival in the 107 patients is shown in Fig. 1. Survival was related to a number of pretreatment variables: the influence of stage B symptoms, gastrointestinal tract involvement, overall assessment of liver function, alkaline phosphatase, serum albumin, histology, peripheral-blood lymphocyte count and haemoglobin were all assessed individually in relation to survival, and following this a Cox regression analysis was performed. Four

TABLE II.—*Multivariate analysis of variables influencing the probability of complete remission in* 107 *patients with lymphomas of diffuse pathology* (*NHD*4/1 *Study, Manchester Lymphoma Group, October* 1980)

| Variable | Scaled deviance | Decrease | P | Favourable feature |
|---|---|---|---|---|
| None | 134·6 | — | | |
| Alkaline phosphatase | 120·7 | 13·9 | 0·0002 | Normal |
| + Age groups | 115·0 | 5·7 | 0·017 | Young |
| + B symptoms | 109·1 | 5·9 | 0·015 | Absent |
| + Marrow | 101·8 | 7·3 | 0·007 | Normal |
| + Sex | 97·7 | 4·1 | 0·043 | Female |

A step-up procedure was used for the analysis.

FIG. 1.—Overall survival of 107 patients with diffuse histiocytic, diffuse poorly differentiated lymphocytic, diffuse mixed and diffuse undifferentiated lymphomas, treated with the VAP protocol.

TABLE III.—*Cox regression analysis of variables influencing survival in* 107 *patients with lymphoma of diffuse pathology*

| Variable | Log likelihood | Increase | P | Unfavourable feature |
|---|---|---|---|---|
| Albumin | 8·45 | 8·45 | 0·00004 | Low albumin |
| + Stage | 12·60 | 4·15 | 0·004 | Stage IV |
| + Alkaline phosphatase | 15·21 | 2·61 | 0·023 | Raised |
| + Pathology | 17·14 | 1·93 | 0·05 | Histology other than diffuse histiocytic |

Each of the 4 variables has a significant effect on survival after adjustment for the other 3.

FIG. 2.—Survival related to stage ($P = 0{\cdot}003$). Stage II patients were a group with abdominal or gastrointestinal involvement, but no evidence of spread outside the abdomen.

FIG. 3.—Survival related to remission status ($P < 0{\cdot}00001$). Complete remission (CR) was defined as resolution of all evidence of lymphoma. Good partial remission (GPR) included patients with > 90% reduction in tumour masses. Partial remission (PR) was defined as > 50% but < 90% reduction in the mass of disease and failure (F) was < 50% reduction in the mass of disease.

variables, including a low serum albumin, Stage IV disease, high serum alkaline phosphatase and diffuse histiocytic pathology, had a significant effect on survival after adjustment for the other 3. None of the other pretreatment variables had a statistically significant effect on survival after adjusting for the effects of these 4 variables (Table III). Patients with abdominal Stage II disease did particularly well under this treatment policy, with more than 70% surviving, relapse-free, beyond 4 years (Fig. 2). Survival was also closely related to remission status which was of individual prognostic significance, with 60% of those achieving a complete remission surviving for more than 4 years. Failure to achieve a complete remission was associated with a poor survival (Fig. 3).

There was a statistically significant difference in survival ($P = 0{\cdot}001$) and relapse-free survival ($P = 0{\cdot}01$) in patients who received bulk radiotherapy after induction chemotherapy. The improved survival seen in patients receiving radiotherapy was still significant after adjustment for the effects of the 4 pretreatment variables in Table III, but this treatment was not randomized, and the data must be interpreted with caution. The results suggest that a prospective randomized study of radiotherapy following induction chemotherapy could be well worthwhile.

Skarin and his colleagues from Boston (Skarin *et al.*, 1980) have recently reported data on another frequent chemotherapy schedule which lends support to the suggestion that this approach may lead to improved complete remission rates and long-term relapse-free survival. A group from Seattle (Sullivan *et al.*, 1979) have also suggested that radiotherapy could have a useful role following initial chemotherapy and one third of the patients completed their remission using radiotherapy in their series. The radiotherapy given included whole-body irradiation if the marrow was involved, and this approach, which was more aggressive than our own, may have contributed to the higher complete-remission rate. The contribution of these different forms of radiotherapy to the improved survival in patients with diffuse lymphomas deserves further study under controlled conditions.

The appreciation that patients with some forms of diffuse pathology have a continuous relapsing pattern and poor survival in spite of a high apparent initial remission rate is disappointing. A more intensive approach using combined chemotherapy and radiotherapy may lead to further improvement in survival, but this remains to be tested. In my view, a radical approach following the induction of remission is now required. In contrast with diffuse histiocytic lymphoma, where high-dose irradiation frequently fails to control local disease, recurrences in the irradiated area after moderate-dose radiotherapy for diffuse lymphocytic or lymphoblastic disease are less common (Fuks & Kaplan, 1975). Low-dose whole-body irradiation (1–3 Gy) has proved of some value in the control of lymphocytic lymphomas, though it is no better than chemotherapy, and myelodepression contributes to difficulties in later treatment (Johnson *et al.*, 1978). For these reasons, an approach using combined intensive combination chemotherapy with higher-dose whole-body irradiation (11 Gy) for selected patients with diffuse poorly differentiated lymphocytic or lymphoblastic forms of lymphoma is now worth studying. The marrow is frequently involved in such patients, and homologous marrow transplantation would be required. The promising results in patients with acute leukaemia using this approach provide a good argument for studying this new method of treatment in patients with diffuse lymphoma in whom a complete remission has been achieved.

### *Central nervous system (CNS) involvement*

Involvement of the CNS is a well known complication in patients with lymphoma. The reported incidence varies from $<5\%$ to $>25\%$, with the highest incidence in patients with diffuse histology. However, few patients are likely to benefit from prophylactic treatment of the CNS, since most patients in whom CNS involvement develops present at a time of advancing or uncontrolled disease in other areas (Young *et al.*, 1979). In addition, the routine prophylaxis may compromise the ability to deliver adequate chemotherapy for cure. For these reasons, the careful selection of patients at high risk is mandatory if prophylaxis is to be tested. Further study of this topic is also warranted because the choice of effective therapy for CNS disease is in doubt. Our policy in the Manchester Lymphoma Group is to avoid prophylactic treatment of the CNS until further data accrue on the incidence of CNS relapse in patients who are otherwise in complete remission.

### CHEMOTHERAPY FOR LOCALIZED DISEASE

Localized or regional radiotherapy may produce prolonged relapse-free survival in about half the patients with lymphomas of diffuse pathology presenting with clinically localized disease (CS I/II). Patients with diffuse histology constitute about 80% of the total presenting with local disease. A recent analysis of data from St Bartholomew's Hospital showed a 50% relapse-free survival for 29 patients with diffuse poorly-differentiated lymphocytic, compared with 34% of 32 patients with diffuse histiocytic histology (Timothy *et al.*, 1979). Their results are similar to other recently published series from Canada (Bush *et al.*, 1979) and the United States (Chen *et al.*, 1979), and are consistent with the earlier literature (see review, Bonadonna *et al.*, 1976). The relapse rates for patients with CS II disease are higher than for CS I disease. Analysis of local failures shows that nearly all involved diffuse tumours and, in contrast with nodular tumours, these can prove difficult to control, even with high-dose irradiation (Fuks & Kaplan, 1975). Nearly all relapses, however, occur by wide dissemination, and chemotherapy therefore has an important role in this context in preventing the growth of tumour in these disseminated sites and the recurrence of tumour in areas treated previously with radiotherapy.

The Milan group have reported their 5-year follow-up data of a controlled randomized trial of combination chemotherapy used as an adjuvant after radiotherapy for pathological stage (PS) I/II NHL (Monfardini *et al.*, 1979). After treatment with regional radiotherapy, patients in complete remission were randomized to receive either no further therapy or 6 cycles of CVP (cyclophosphamide, vincristine and prednisolone). A total of 96 patients were evaluable. At 5 years from completion of irradiation, the relapse-free survival was 46% after radiotherapy and 72% after radiotherapy with CVP ($P = 0{\cdot}005$). The corresponding findings for the *overall* survival calculated from the *beginning* of irradiation were 55% and 83% respectively ($P = 0{\cdot}03$). The favourable effect of adjuvant chemotherapy on relapse-free survival was statistically significant, irrespective of stage and clinical presentation in the subgroup with diffuse histology which represented more than 70% of the entire series. In contradistinction, patients with nodular histology showed no improved relapse-free survival after 6 cycles of CVP. In patients relapsing after radiotherapy alone, salvage therapy failed to induce a high incidence of second durable remissions. This study is important in that 98% of the patients had pathological staging (26% by laparoscopy and 74% by laparotomy). Glatstein and his colleagues from Stanford (Glatstein *et al.*, 1977) reported a study in which they failed to observe an improvement in overall survival or relapse-free survival in PS I/II patients with high-grade histology using total nodal irradiation with CAT (cytosine arabinoside, Adriamycin and thioguanine) for diffuse histiocytic lymphoma, and CVP for all other histologies. This was also true for the study reported by Panahon *et al.* (1977). The reason for these different results may well be related to the differences in the radiotherapy rather than the chemotherapy used in the studies.

A study from Stockholm supports the findings of the Milan group (Landberg *et al.*, 1979). Fifty five patients with nodular or diffuse lymphoma of CS I/II were randomized for 9 cycles of CVP after radiotherapy; the relapse-free survival at 30 months was 41% for patients without and 86% for patients with adjuvant chemotherapy ($P = 0{\cdot}02$). Survival was the same for both treatment arms, being 90% at 30 months. Analysis of the subgroups showed that adjuvant chemotherapy significantly prolonged the relapse-free survival in diffuse histiocytic lymphoma, but there were only 20 evaluable patients in this group. This study suffers from the defect of small numbers of patients and short follow-up.

The Manchester Lymphoma Group is conducting a study comparing the effects of two forms of combination chemotherapy as an adjuvant after radiotherapy for nodal stages I/II disease. Patients with Stage $I_E$ disease were not included since these have a relatively good relapse-free survival after radiotherapy alone. The chemotherapy was either 6 courses of a combination without Adriamycin (CMOPP—cyclophosphamide, vincristine, procarbazine and prednisolone) or the regimen used by the group for advanced stages of high-grade lymphoma (vincristine, Adriamycin and prednisolone for 6 weeks followed by 2 years oral 6-mercaptopurine, cyclophosphamide and Methotrexate). Only 5/34 patients have relapsed (median follow-up $2\frac{1}{2}$ years) with a 3-year relapse-free survival of 88%. As yet, there is no significant difference between the treatment arms, since the number of events is too low.

The recognition that chemotherapy alone can produce long-term relapse-free survival in patients with Stage III/IV diffuse histiocytic lymphoma, and that adjuvant chemotherapy prolongs survival *and* relapse-free survival in carefully staged localized diffuse histiocytic lymphoma has prompted the use of chemotherapy alone in patients with Stage I/II lymphoma of this type. Miller & Jones (1979) retrospectively analysed a series of 22 patients with diffuse lymphoma, Stages I/II, treated with chemotherapy alone (14 patients) or chemotherapy with local irradiation. All 22 patients achieved a complete remission and remained alive (median survival 27+ months). Twenty one patients remained continuously free of disease, with a median relapse-free survival from completion of chemotherapy of 23+ months. Most patients received CHOP chemotherapy (cyclophosphamide, Adriamycin, vincristine and prednisolone).

It seems that chemotherapy now has an established role in the treatment of Stage I/II lymphomas of high-grade histology.

### STAGING

The studies I have already mentioned emphasize the importance of staging. A further example of the important role of careful staging can be obtained from a study of patients presenting with gastrointestinal lymphoma. A recent retrospective series of patients with gastrointestinal lymphoma presenting at the Christie Hospital, Manchester, has been analysed by our group (Blackledge *et al.*, 1979). There were 104 patients with full details of the surgery obtained. Although the median survival was only 15 months, 35% were alive and well at 10 years. The tumour type (histology, site and whether single or multiple) extent of lymph-node involvement and the presence of local extension to adjacent organs, perforation with peritonitis or distant metastases were of considerable prognostic importance. The Ann Arbor staging classification was inadequate for this group of patients, and a new staging system for gastrointestinal lymphoma has been proposed, which has prognostic significance and can be used to select appropriate poor prognostic groups for chemotherapy. It was clear from the data that patients fared much better if tumour excision was complete. Each of the patients had initial surgery involving laparotomy, and all apparent tumour was removed in 41 patients. This included removal of locally involved nodal masses and tumour which had spread to adjacent tissues. The group had a much better survival than the remainder with either incomplete tumour removal or merely tumour biopsies ($P = 0{\cdot}0005$). Of the 49 patients who had complete removal of the tumour, only 11 had a single tumour confined to the gut; all the others had either local nodes involved or spread to adjacent tissues. Considering only Stage II patients with disease extending outside the gastrointestinal tract, there was still a highly significant difference between the patients with complete and incomplete removal of the tumour ($P = 0{\cdot}004$). Cure is a distinct possibility after surgery alone for patients with gastrointestinal lymphoma, but tumour removal must be complete. Other groups with incomplete removal have a poor survival, in spite of the addition of radiotherapy, and early chemotherapy is then of recognizable value.

Unlike most forms of nodal lymphoma, gastrointestinal lymphoma has a propensity for remaining apparently localized to the gut wall and draining lymph nodes (gastric or mesenteric) allowing a moderate proportion of cures by surgery alone. Peripheral nodes become palpable late in the history of the disease, if at all, and in this series, only two patients had a palpable spleen at presentation. Only 20% had widespread nodal disease; involvement of adjacent organs after spread through the bowels was more common than indirect metastatic spread to distant organs, and the bulk of disease remains confined to the abdomen for most of its course. For these reasons, the Ann Arbor classification, which is so useful for nodal Hodgkin's disease, is less appropriate for gastrointestinal NHL. A study of the pattern of lymphoid-cell migration can offer an explanation of the difference between gastro-intestinal lymphoma and other forms of lymphoma, and will be discussed later.

*Whole-body scanning with computed tomography (CT)*

Surgical staging is not recommended for most patients presenting with NHL, since laparotomy is potentially dangerous and generalized disease can usually be documented by conventional clinical staging. Treatment decisions are usually based on staging procedures which avoid laparotomy in this group of patients. CT has enabled a more accurate documentation of the pattern of disease at presentation than could be achieved by conventional clinical staging alone, and therefore plays an important part in initial staging. Its advantages over abdominal lymphography have previously been documented (Crowther *et al.*, 1979). In Manchester, whole-body CT has replaced abdominal lymphography in lymphoma patients.

At presentation and clinical relapse, CT scanning detects unsuspected disease in a high proportion of patients with lymphoma, and has an important influence on treatment policy. The evaluation of response to chemotherapy and the detection

TABLE IV.—*Abdominal CT scan results in patients with diffuse-pathology lymphoma in apparent clinical remission by conventional restaging methods (excluding lymphography)*

| | No. | Alive relapse-free | Relapsed or dead |
|---|---|---|---|
| CT normal | 22 | 21 | 1 |
| CT abnormal | 21 | 5 | 16 |

$P = 0{\cdot}00001$.

of bulk disease for subsequent radiotherapy are of additional importance in patient management, and the technique is of great value in documenting remission. Preliminary data on the importance of assessing remission status after treatment and the value of CT in this context has previously been published (Best *et al.*, 1978). An update of these results in lymphomas with histology of diffuse large-cell type is shown in Table IV. Forty-three patients had abdominal CT scans when they were considered to be in complete clinical remission after chemotherapy. Conventional restaging methods were used with documentation of remission, using biochemical, haematological (including marrow aspiration and trephine assessment), cerebro-spinal fluid examination and conventional radiology (excluding lymphography and CT scanning). All patients showed resolution of symptoms and signs of pre-existing lymphoma. Of the 43 patients, 21 had abnormal CT scans, and 16 of these have relapsed or died of lymphoma. Of the 5 with abnormal scans who are alive and well, 2 who showed persistant disease within 2 months of clinical remission had a regression with further treatment over the ensuing months. Of the 22 patients with normal scans, only 1 has relapsed. This study emphasizes the importance of carefully documenting remission status in these patients, and its reflection on survival has been shown in Manchester Lymphoma Group data (Fig. 3).

In passing, it must be said that results in patients with nodular lymphoma were different, and showed no difference in relapse-free survival or overall survival between those with abnormal scans and patients with no evidence of disease on CT scans (unpublished data).

An accurate documentation of sites of relapse is important in deciding the most appropriate therapy for patients with evidence of recurrence. CT has proved to be extremely helpful in this respect. Of 27 patients relapsing with diffuse histology, 23 had abnormal CT scans, and CT detected about twice as many areas of involvement as was expected by conventional clinical restaging. Table V shows details of sites involved in patients with nodular and diffuse pathologies. Lymphography was not performed routinely in this group as a relapse investigation, but it is likely that, of the 50 unexpected lymph-node areas that were shown to be involved by CT, lymphography would have detected only 11 (23%) of them, these being in the iliac and mid or lower para-aortic regions. All but 3 of the 15 retrocrural abnormalities were unsuspected clinically, and coeliac or mesenteric nodes were rarely suspected by conventional restaging, though they were seen to be enlarged in 19 cases using CT.

TABLE V.—*Abdominal sites involved in patients with relapsing lymphoma detected by CT (50 patients)*

| | Nodular histology | | Diffuse histology | |
|---|---|---|---|---|
| Patient number | 23 | | 27 | |
| Abdominal CT scan abnormal | 18 | | 23 | |
| | Clinically | | Clinically | |
| Areas involved | Expected | Unexpected | Expected | Unexpected |
| Retro-crural node | 0 | 7 | 3 | 5 |
| Para-aortic node | 13 | 7 | 6 | 7 |
| Iliac nodes | 9 | 3 | 7 | 1 |
| Coeliac nodes | 0 | 5 | 0 | 4 |
| Mesenteric nodes | 1 | 5 | 0 | 4 |
| Liver | 6 | 3 | 5 | 1 |
| Spleen | 5 | 4 | 4 | 3 |
| Splenic hilar node | 0 | 1 | 0 | 1 |
| Other areas | 4 | 3 | 7 | 0 |
| Total | 38 | 38 | 32 | 26 |

HUMAN LYMPHOCYTE TRAFFIC

I would now like to turn to a topic that has interested my group for the last few years; namely lymphoid cell traffic in man and its relationship to the behaviour of malignant lymphoid cells *in vivo*. The studies in man were largely performed by Dr John Wagstaff in my department, but important contributions to their success have been made by Mr C. Gibson, Dr N. Thatcher and Professor W. Ford (Department of Experimental Pathology, Manchester University).

The migratory pattern of different normal lymphocyte populations is known to be of fundamental importance in the development of an immune response. Although the migration of lymphocytes from blood to the tissues and their return has been well established in small experimental animals, using autoradiographic techniques and cannulation of lymphatics, data in humans are sparse. A study of the migration of clones of malignant lymphoid cells in both animals and man could well lead to a better understanding of the physiology of normal lymphocyte migration and help to explain the pattern of distribution in malignancies. Such studies are analagous to the studies of inherited cellular immune deficiencies which help to dissect the mechanism of the immune response in man.

It has been observed in rats with Roser leukaemia that coeliac nodes enlarge by a factor averaging over 500 whereas superficial cervical lymph nodes undergo only a 4-fold enlargement. Further study has shown that preferential migration of lymphoma cells from the blood to the coeliac node in this condition contributed to the unusual pattern of distribution (Ford, 1978). Lymphoid blast cells arising from stimulation with an allogeneic graft, however, also show a predilection for the coeliac node, but in this situation the node does not become excessively large, since the cells have a short transit time (Smith *et al.*, 1980). Preferential migration and alterations in transit time may therefore be of major importance in determining a pattern of disease distribution. Ford has suggested that the high incidence of coeliac-node involvement in Hodgkin's disease could be explained by migration of tumour cells *via* the blood to the liver and thence to the coeliac node, rather than by retrograde lymphatic spread. In addition, the use of lymphoma lines in experimental systems has indicated that there may be differences in the high endothelial venules of different tissues and organs through which lymphocytes migrate (Butcher & Weissman, 1980).

Surface-marker and enzyme studies have shown that the malignant cell in most NHL patients is of lymphocyte lineage. A study of the migratory behaviour of these cells is therefore likely to be of importance, not only in understanding more about the nature of the immune deficiency in these patients, but also may help explain the pattern of spread in the various types of malignant lymphoma. For example, an understanding of the migratory behaviour of lymphoid cells offers an explanation of the disease distribution pattern seen in the gastrointestinal lymphomas, where the main bulk of disease remains confined to the gut and mesenteric node areas for a considerable period in an appreciable proportion of patients. It is now recognized that lymphocytes from gut-associated lymphoid tissue have characteristic recirculation patterns (see review by Hall, 1980). Immunoblasts and small lymphocytes obtained from efferent lymph draining the small intestine, preferentially home to regions adjacent to the lamina propria or to the small intestine (very few to peripheral nodes or to the large intestine). Immunoblasts and small lymphocytes obtained from efferent lymph draining peripheral nodes, on the other hand, preferentially home to peripheral nodes and spleen. Both T and B cells have subpopulations with migratory characteristics of peripheral nodal or intestinal type. It is to be expected that if the phenotype responsible for this migration pattern is conserved in patients with lymphomas arising from gut-associated lymphoid cells, the bulk of tumour would be confined to those areas for a prolonged period in spite of blood involvement.

Many of the techniques used for the study of lymphocyte migration in experimental animals are invasive and impossible to carry out in man. The few human studies have mainly used $Na_2{}^{51}CrO_4$ labelled lymphocytes; a technique with many disadvantages. McAfee & Thakur (1976) showed that Indium-111 oxine conjugate was an efficient means of labelling cells *in vitro*, and studies using Indium-111 oxine as a lymphocyte label have demonstrated that more reliable information on lymphocyte kinetics in animals systems can be obtained by this method (Rannie *et al.*, 1977*a,b*; Chisholm *et al.*, 1978; Issekutz *et al.*, 1980; Sparshott *et al.*, in preparation). Lavender *et al.* (1977) showed that external imaging of $^{111}$In-labelled lymphocytes was possible in man, and following studies in Manchester, the method has an established value in following the traffic of normal lymphocytes and their malignant counterparts in man (Wagstaff *et al.*, 1981*a,b*).

Indium-111 oxine is a lipid-soluble complex with a high labelling efficiency, a low elution rate, and produces Auger electrons which allow autoradiography. The gamma emission spectrum is ideal for external imaging on a conventional gamma camera. By using a combination of gamma-camera imaging and surface-probe counting, it is possible to assess the changing patterns of distribution of lymphocytes in man following re-injection.

When the lymphocyte population from control subjects (predominantly T cells) is labelled and re-injected, the number of labelled cells in the blood falls during the first 4 h. The cells leaving the blood mainly accumulate in the spleen, which shows increased imaging during this initial period. There is an increase in labelled cells in the blood during the 4–24 h period during which the splenic activity falls by about 40%. During this period the cells accumulate in lymph nodes. The transit

FIG. 4.—Blood-clearance curves, together with spleen and liver uptake, of indium-111 oxine-labelled lymphocytes from a normal subject. The secondary rise of labelled lymphocytes in the blood (at 4 h) is seen at a time when the surface probe counts over the spleen are decreasing.

time of small lymphocytes through the human spleen seems to be similar to that in small experimental animals, and preliminary data suggest that the traffic into and out of lymph nodes also approximates to that found in experimental animal systems. The data are consistent with the T cells leaving the blood and entering the spleen after re-injection. After 4–6 h, they would have traversed the splenic white pulp and reappeared in the blood, causing the observed secondary rise in the blood-clearance curves (Fig. 4).

The transit of different lymphocyte subpopulations in the blood make an important contribution to the magnitude of the immune response *in vivo*, and quantitative aspects of lymphocyte traffic are important in the interpretation of *in vitro* immunological studies of lymphoid cells taken from the blood. Much less time is spent in the blood than in the other tissues where immunological reactions take place, and the relative numbers of lymphocytes measured in the blood with different functional characteristics may not reflect the magnitude of the immune response.

Patients with a monoclonal expansion of the B-cell subtype in the peripheral blood show a different distribution pattern of re-injected lymphocytes from normal controls, or from lymphoma patients with no apparent monoclonal B-cell expansion in the blood. Seven patients with chronic lymphocytic leukaemia (CLL) have been studied and all showed a rapid exponential decrease in the percentage of labelled lymphocytes in the blood volume (Fig. 5) (Wagstaff *et al.*, unpublished). Unlike controls, CLL patients showed no evidence of a rise of labelled cells in the blood in the 4–24h period. Patients with CLL or lymphomas with B lymphocytosis showed a continuous rise in uptake by the spleen between 4 and 24 h, with a fall between 24 and 48 h. This contrasts with the fall in counts over the spleen 4 h after the re-injection of normal lymphocytes into control subjects. The more continuous removal of labelled B cells from the blood in the patients with B-cell malignancy sug-

FIG. 5.—Blood-clearance curves, together with spleen and liver uptake of indium-111 oxine-labelled lymphocytes from a patient with chronic lymphocytic leukaemia. Unlike normal lymphocytes (Fig. 4) there is no secondary increase of labelled cell sin the blood following their rapid clearance, and the probe counts over the spleen are relatively constant over 48 h.

gests that even at 48 h, few labelled cells have returned to the blood after primary localization. The distribution to heavily involved organs such as the liver and marrow in these patients has also been observed. Marrow imaging is more intense in CLL patients than in control subjects.

It does not follow that the kinetics of migration of these cells is related to their malignancy, however, since it is known that B cells in animals have a more prolonged migration pattern than T cells (Ford, 1975—review). T lymphocytes generally recirculate much more rapidly than B cells, though they leave the blood by crossing the post-capillary venules in lymph nodes at the same rate. T cells have a mean transit time of 5–6 h through the spleen, and 16–18 h through lymph nodes, compared with B cells which have not left the spleen in significant numbers by 24 h after re-injection and have a mean transit time of 30–36 h through the lymph nodes. The observed difference in distribution seen in patients with lymphoid-cell maliglignancy could be explained by the proportion of T and B cells used, since T cells migrate more rapidly through the spleen, the major site of primary localization of lymphocytes. The subsequent rise and plateau seen under normal circumstances could be due to the reappearance of labelled T cells in the blood that have passed through the spleen. The question of whether CLL B lymphocytes have migratory properties similar to normal B cells needs to be answered. This should be possible with further work using the $^{111}$In-oxine labelled lymphocytes.

Other subsets of lymphocytes within the blood are known to have different patterns of migration in experimental systems, and gut, lung and salivary lymphoid tissue may well have characteristic migratory patterns. Further work on this important new concept, using experimental animals, would be well worthwhile.

Using $^{111}$In oxine-labelled cells, it can be shown that some patients with other malignancies have an extremely large flux of lymphocytes through tumour tissue.

Fig. 6, for example, shows a scan of the chest in a patient with nodular sclerosing Hodgkin's disease involving the mediastinum a few hours after injecting his own labelled lymphocytes. A very large number of labelled lymphoid cells has been taken up by the mediastinal tumour, and visualization of the enlarged nodes may occur as early as 30 min after re-injection. Such a flux of lymphocytes may be an important mechanism enabling contact to be made between tumour tissue and the reactive lymphoid cells which are known to be in the blood of patients with Hodgkin's disease (Crowther *et al.*, 1969*a*,*b*). These cells are similar to those seen in the blood after antigenic stimulation.

(a)

(b)

FIG. 6.—(a) A plain chest X-ray showing mediastinal enlargement with nodular sclerosing Hodgkin's disease. (b) Gamma-camera picture 24 h after the patient's own peripheral-blood lymphoctyes labelled with indium-111 oxine were re-injected.

Although an antigenic stimulus promotes lymphocyte migration, antigen specificity does not appear to contribute to the initial homing. Lymphocyte migration is, however, facilitated by an increase in local blood flow, and this appears to be an important mechanism for bringing antigen-specific lymphoid cells into appropriate contact with foreign material at sites of inflammation (Hay *et al.*, 1980).

We are also hoping that the technique will shed light on the cause of the lymphopaenia in patients with malignant disease and its relationship with prognosis.

### CLASSIFICATION

Classification of NHL by conventional histopathological techniques is notoriously unreliable, and the large number of histopathological classifications available reflects this problem. The cell types can be described, but the cell lineage and differentiation cannot be determined without more sophisticated techniques.

Normal lymphoid cells and their malignant counterparts have been reported to vary in their biochemical properties, and variation in surface glycoproteins, membrane-transport molecules, content of metabolites such as glycogen, ability to synthesize certain amino acids and specific enzyme content has been observed. Biochemical characterization is proving of objective value in classifying lymphoproliferative tumours in addition to routine histological techniques. Heterogeneity of lymphoid malignancies has been demonstrated within the T-cell, B-cell and non-T, non-B groupings with respect to surface properties of the individual cells, and enzymatic content and correlations with clinical features have been noted.

The classification of lymphoproliferative disorders has dramatically changed in the last few years, as our understanding of immunology has increased. Studies of membrane properties and functional attributes of the lymphocytes has led to a better understanding of the biology of the lymphomas. Subpopulations of both T and B cells have been identified, and characteristic lymphoma patterns can be related to a homogeneous expansion of a particular subpopulation. In addition, immunological studies are providing information on the sequence of cell-surface-related differentiation in cells of the lymphocytic, granulocytic and erythrocytic series.

The method of monoclonal antibody production using mouse hybridomas (Kohler & Milstein, 1975) is already proving of value, in addition to the more generally available techniques for characterizing cell-surface phenotypes within the lymphoid malignancies. The biochemical features of the antigenic determinants reacting with monoclonal antibodies can now be investigated, and this most important area of research is now open. Monoclonal antibodies may be directed against specific oligosaccharide sequences, and can be used to dissect the chemistry of the cell-surface glycoproteins. Preliminary results suggest that the technique is of importance in determining the immunological phenotype of patients with diffuse large-cell lymphoma, and this may be helpful in defining groups with different survival prospects (Warnke *et al.*, 1980). Clearly, prospective studies involving these new techniques in untreated patients are likely to lead to a better understanding of the biology of these tumours, but the prognostic weight of each parameter must be measured against other known prognostic features, using multi-variate analysis. An approach has already been made to evaluate enzyme and membrane marker properties of leukaemia cells using multi-parameter analysis (Janossy *et al.*, 1980).

The detection of cells of B lineage belonging to a single clone in the peripheral blood of patients with lymphoma can help confirm the diagnosis and define the tumour type. Such studies also have relevance in terms of staging (Garrett *et al.*, 1979; Ault *et al.*, 1979). Abnormalities can be detected in the presence of a normal white cell count and differential using anti-light-chain sera, but interpretation is dependent on the ratio of Kappa: Lambda bearing cells. Other techniques, such as the detection of colchicine-sensitive lymphoid cells in the peripheral blood may also be helpful in this regard (Thomson *et al.*, 1972; Scarffe *et al.*, 1980).

These methods, however, are not the only ones available for studying the composition of the plasma membrane in single cells. A method of current interest to our group in Manchester involves a study of the biochemistry of the lymphoid cell surface using plant-lectin binding assessed by flow cytometry. A large number of workers are contributing to the success of these studies, but particular mention must be made of Dr G. Blackledge who conducted the flow-cytometric studies and Dr J. Gallagher who provided the necessary expertise in glycoprotein and lectin chemistry. The immunological studies were in collaboration with Dr B. Vose (Department of Immunology, Paterson Laboratories).

Lectins can be bound to fluorescein isothiocyanate, and the amount attaching to the plasma membrane of single cells can be determined by measuring the emission of fluorescence from each cell in flow cytometry. Differences in lectin binding between cells within a separated population may be investigated using this method. Flow cytometry involves the measurement of different properties of cells passing singly at high speed through a beam of light (Kamentsky *et al.*, 1965). Light scattering of the beam (related to cell size) and emission of light from a specific fluorescent probe can be assessed quantitatively for each cell. An interface between the flow cytometer and a Desk Top computer (HP9845S) allows a 3-dimensional presentation of data for cell size, number and fluorescence measurements (Blackledge *et al.*, 1980*a*). Cell populations with different properties can easily be recognized and selected for further study, using this system. The method is rapid and means that the lectin-binding properties of several thousand individual cells within a population can be characterized.

The carbohydrate residues of glycoproteins and glycolipids are located on the external face of the plasma membrane, where they play an important part in cell behaviour. The migratory properties of normal lymphoid cells and their malignant counterparts may well be determined to a large extent by differences in cell-surface chemistry. For these reasons, the study of certain plant lectins with highly specific saccharide-binding properties is being undertaken. The technique with flow cytometry provides a tool for examining the carbohydrate structure of the intact plasma membrane of single cells.

Some lectins react only with certain terminal sugar residues, others react with sugars within the carbohydrate chain, and occasionally they may react specifically with a particular sugar sequence. The use of enzymes to remove terminal sugar residues, followed by further binding studies with appropriate lectins, will allow the concentration and/or affinity of specific subterminal sugars to be determined.

When a lymphoid cell population is obtained from peripheral blood by removing the erythrocytes and phagocytic cells, the lymphocytes show a characteristic pattern of binding with 60% of the cells having low binding to *Lens culinaris* agglutinin (LCA) and the remaining 40% showing considerably higher binding, independent of cell size. This plant lectin binds to α-mannoside residues within the carbohydrate chain, and binding can be inhibited by the analogue α-methyl mannoside. Separation of the T lymphocytes on the basis of the capacity to form rosettes with sheep red blood cells ($E^+$) indicated that most (80–87%) had low LCA binding, whereas lymphocytes not binding to sheep red blood cells ($E^-$) had high binding activity. The production of an enriched T-cell population by removing B cells in a bead column coated with human Ig reacted with human anti-Ig, produced a similar binding pattern to the T-cell population separated with sheep red cells. Subsequent experiments using a Beckton Dickinson Fluorescence-activated Cell Sorter 4, showed that $E^+$ cells could be separated on the basis of LCA binding (Blackledge *et al.*, in preparation). Study of the patterns of inhibition with appropriate sugars indicate that the distinction between T ($E^+$) and non-T ($E^-$) cells is quantitative rather than qualitative, with non-T cells having the greater number of both high- and low-affinity sites for LCA. This means that the concentration of N-glycosidically-linked mannose containing oligosaccharides is highest in the $E^-$ population. Wheat germ agglutinin (WGA) binds to terminal sialic acid and N-acetyl glucosamine residues and can be used to indicate the number of complete cell-surface oligosaccharides. Experiments with this lectin show increased binding in the $E^-$ subset, indicating a higher saccharide chain density in these cells.

Further experiments, in which subsets of lymphocytes with different functional characteristics were separated by a density-gradient technique, have allowed a correlation to be made between functional activity and lectin-binding properties (Vose *et al.*, in preparation). Studies involving monoclonal antibodies and lectin binding are providing the tools for separating subsets of lymphocytes with different functional capacity, and provide a new approach in removing unwanted immune cells from a marrow graft.

Cell-surface lectin-binding properties of malignant lymphoid cells can be studied by similar techniques. The lectin-binding properties of acute leukaemia cells taken from the peripheral blood differ markedly from those of a normal peripheral-blood lymphocyte population, and cells of myeloid and lymphoid lineage appear to have distinctive features (Fig. 7). The normal lymphocyte population is heterogeneous in lectin-binding properties, whilst the leukaemia-cell population is homogeneous. In the example shown in Fig. 7, the ALL cells show low concanavalin A (Con A), LCA and WGA binding, whereas the AML cells show much greater binding to these lectins.

Studies of lymphoid cell populations in the peripheral blood from patients with lymphomas (Fig. 8) shows the marked heterogeneity of the blood lymphoid-cell populations from lymphoma patients in terms of lectin binding. The binding pattern to Con A, LCA and WGA is quite different from that of the normal lymphocyte population, and may indicate peripheral-blood involvement in the different lymphomas. The binding properties can return to normal after induction of remission, and the study of binding profiles could play a part in the continuing evaluation of disease. Work is continuing in characterizing clones of malignant cells from patients with lymphoma in terms of lectin binding and surface chemistry, and we hope to relate this to their migratory properties and the behaviour of the tumour in the patient.

FIG. 7.—Lectin-binding profiles of normal peripheral-blood lymphocytes compared with acute-leukaemia cells. A double peak in *Lens culinaris* agglutinin (LCA) binding is seen in normal peripheral-blood lymphocytes, compared to a single peak in the more homogeneous sample of leukaemia cells. Acute lymphoblastic leukaemia (ALL) cells show low binding, whereas acute myeloblastic leukaemia (AML) cells show high binding of all 3 lectins. Peripheral-blood lymphoid cells from the patient with Hodgkin's disease (HD) show heterogeneity, with many of the cells showing very low Concanavalin A (Con A) binding, but a small population showing markedly increased LCA and Wheat Germ Agglutinin (WGA).

FIG. 8.—Lectin-binding profiles of peripheral-blood lymphoid cells from patients with non-Hodgkin's lymphoma. These samples from patients with diffuse histiocytic lymphoma (DHL), diffuse poorly differentiated lymphocytic (DPDL), nodular poorly differentiated lymphocytic (NPDL) and nodular well differentiated lymphocytic (NWDL) lymphomas show the heterogeneity in lectin binding compared with the normal lymphocytic population (Fig. 7); several cell populations are visible.

The abnormal lectin binding of lymphoid cells from the peripheral blood of patients with Hodgkin's disease is of interest (Fig. 7). Reactive lymphoid cells are known to be increased in the peripheral blood of such patients, and it is tempting to surmise that the markedly increased LCA and WGA binding seen in a proportion of the cells may be related to this. The large number of cells with very low Con A binding is also an interesting feature, and requires further study.

A further technique involving the assessment of lectin binding by fluorescence microscopy of lymph-node sections has been devised, and this is providing useful information on lectin binding to the plasma and nuclear membrane of normal lymphoid cells and malignant lymphoma cells. In these studies the lymph-node architecture remains intact and the work is providing useful additional information about differences between lymphomas (Bramwell *et al.*, unpublished observations).

I started my talk by illustrating the lack of improvement in overall survival in patients with NHL in England and Wales during the 1960s and early 1970s. However, I hope I have demonstrated that there is no need for pessimism. The improved combined-modality treatment already available for the least favourable group of patients should now lead to some improvement in these national figures. The intensity of research into distinguishing groups of patients with characteristic malignant cells and biological behaviour is leading to innovative approaches to therapy likely to improve the welfare of patients in the poor-risk groups. The paths of basic research and clinical research are closer in this subject than they have ever been before, and it is clear that the 1980s will be coloured by further exciting joint ventures of value to the patient.

I would like to thank my colleagues in the Manchester Lymphoma Group for allowing me to publish the data concerning chemotherapy in the non-Hodgkin's lymphoma of diffuse pathology. Dr M. Palmer, Dr G. Blackledge, Mr R. Swindell and Dr V. Blair were responsible for the development of the computerized analysis presented here. I am most grateful to my secretary, Mrs B. Whittle, for typing the manuscript.

### REFERENCES

AULT, K. A. (1979) Detection of small numbers of monoclonal B lymphocytes in the blood of patients with lymphoma. *N. Engl. J. Med.*, **300**, 1401.

BEST, J. J. K., BLACKLEDGE, G., FORBES, W. ST C. & 4 others (1978) Computed tomography of abdomen in staging and clinical management of lymphoma. *Br. Med. J.*, ii, 1675.

BLACKLEDGE, G., BUSH, H., DODGE, O. G. & CROWTHER, D. (1979) A study of gastro-intestinal lymphoma. *Clin. Oncol.*, **5**, 209.

BLACKLEDGE, G., SWINDELL, R., HODGSON, B. & CROWTHER, D. (1980*a*) Computerised acquisition and analysis of flow cytometric data. *Int. J. Bio-Med. Comput.*, **11**, 41.

BLACKLEDGE, G., BUSH, H., CHANG, J. & 9 others (1980*b*) Intensive combination chemotherapy with Vincristine, Adriamycin and Prednisolone (VAP) in the treatment of diffuse histology non-Hodgkin's lymphoma. *Eur. J. Cancer*, **16**, 1459.

BODEY, G. P. & RODRIGUEZ, V. (1978) Malignant lymphoma. *Sem. Haematol.*, **15**, 221.

BONADONNA, G., LATTUADA, A. & BANFI, E. (1976) Recent trends in the medical treatment of non-Hodgkin's lymphoma. *Eur. J. Cancer*, **12**, 661.

BUSH, R. S., GOSPODAROWICZ, M., BERGSAGEL, D. E. & BROWN, T. C. (1979) Radiation therapy for patients with localised non-Hodgkin's lymphoma. In *Advances in Medical Oncology, Research and Education. Leukaemia & Lymphoma.* Ed. Crowther. Oxford: Pergamon Press. p. 209.

BUTCHER, E. & WEISSMAN, I. (1980) I. Cellular, genetic and evolutionary aspects of lymphocyte interactions with high endothelial venule. In *Blood Cells and Vessel Walls: Functional Interactions.* Ed. O'Connor. London: Churchill. p. 265.

CABANILLAS, F., RODRIGUEZ, V. & FREIREICH, E. J. (1978) Improvement in complete response rate, duration of response and survival with Adriamycin combination chemotherapy for non-Hodgkin lymphomas: A prognostic factor comparison of two regimes. *Med. Paediat. Oncol.*, **4**, 321.

CHEN, M. G., PROSNITZ, L. R., GONZALEZ-SERVA, A. & FISCHER, D. B. (1979) Results of radiotherapy in control of Stage I and II non-Hodgkin's lymphoma. *Cancer*, **43**, 1245.

CHISHOLM, P. M. & FORD, W. L. (1978) Selection of antigen-specific cells by adherence to allogeneic cell monolayers: Cytolytic activity, graft-versus-host activity and numbers of adherent and non-adherent cells. *Eur. J. Immunol.*, **8**, 438.

CROWTHER, D., BLACKLEDGE, G. & BEST, J. K. (1979) The role of computed tomography of the abdomen in the diagnosis and staging of patients with lymphoma. *Clin. Haematol.*, **8**, 567.

CROWTHER, D., HAMILTON-FAIRLEY, G. & SEWELL, R. L. (1969*a*) Lymphoid cellular responses in the blood following immunisation in man. *J. Exp. Med.*, **129**, 849.

CROWTHER, D., HAMILTON-FAIRLEY, G. & SEWELL, R. L. (1969*b*) The significance of the changes in circulating lymphoid cells in Hodgkin's disease. *Br. J. Med.*, **2**, 473.

FISHER, R. I., DE VITA, V. T., JOHNSON, B. L., SIMON, R. & YOUNG, R. C. (1977) Prognostic factors for advanced diffuse histiocytic lymphoma following treatment with combination chemotherapy. *Am. J. Med.*, **63**, 177.

FORD, W. L. (1975) Lymphocyte migration and the immune responses. *Prog. Allergy*, **19**, 1.

FORD, W. L. (1978) Malignant lymphoma. *UICC Tech. Rep. Series*, **37**, 206.

FUKS, Z. & KAPLAN, H. S. (1975) Recurrence rates following radiation therapy of nodular and diffuse malignant lymphoma. *Radiology*, **108**, 675.

GARRETT, J. V., SCARFFE, J. H. & NEWTON, R. K. (1979) Abnormal peripheral blood lymphocytes and bone marrow infiltration in non-Hodgkin's lymphoma. *Br. J. Haematol.*, **42**, 41.

GLATSTEIN, E., DONALDSON, S. S., ROSENBERG, S. A. & KAPLAN, H. S. (1977) Combined modality therapy in malignant lymphomas. *Cancer Treat. Rep.*, **61**, 1199.

HALL, J. G. (1980) An essay on lymphocyte circulation and the gut. *Monogr. Allergy*, **16**, 100.

HAY, J. B., JOHNSTON, M. G., VADAS, P., CHIN, W., ISSEKUTZ, T. & MOVAT, H. Z. (1980) Relationships between changes in blood flow and lymphocyte migration induced by antigen. *Monogr. Allergy*, **16**, 112.

ISSEKUTZ, T., CHIN, W. & HAY, J. B. (1980) Measurement of lymphocyte traffic with Indium-111. *Clin. Exp. Immunol.*, **39**, 215.

JANOSSY G., HOFFBRAND, A. V. GREAVES M. F. & 6 others (1980) Terminal transferase enzyme assay and immunological membrane markers in the diagnosis of leukaemia: A multiparameter analysis of 300 cases. *Br. J. Haematol.*, **44**, 221.

JOHNSON, R. E., CANELLOS, G. P., YOUNG, R. C., CHABNER B. A. & DE VITA, V. T. (1978) Chemotherapy (cyclophosphamide, vincristine and prednisone) versus radiotherapy (total body irradiation) for Stage III–IV poorly differentiated lymphocytic lymphoma. *Cancer Treat. Rep.*, **62**, 321.

JOHNSON, G. J., COSTELLO, W. G., OKEN, M. M. & 5 others (1979) The value of Adriamycin and of mid-cycle treatment in multidrug therapy of unfavourable histology non-Hodgkin's lymphoma. *Proc. Am. Soc. Clin. Oncol.*, **20**, 196.

JONES, S. E., GROZEA, P. N., METZ E. N. & 6 others (1979) Superiority of Adriamycin-containing combination chemotherapy in the treatment of diffuse lymphoma. *Cancer*, **43**, 417.

KAMENTSKY, L. A., MELAMED, M. R. & DORRAN, H. (1965) Spectrophotometer: New instrument for ultrarapid cell analysis. *Science*, **150**, 630.

KOHLER, G. & MILSTEIN, C. (1975) Continuous cultures of fused cells secreting antibody of predefined specificity. *Nature*, **256**, 495.

LANDBERG, T. G., HAKANSSON, L. G., MOLLER, T. R. & 8 others (1979) CVP-remission-maintenance in Stage I or II non-Hodgkin's lymphoma. Preliminary results of a randomised study. *Cancer*, **44**, 831.

LAVENDER, J. P., GOLDMAN, J. M., ARNOT, R. N. & THAKUR, M. L. (1977) Kinetics of Indium-111 labelled lymphocytes in normal subjects and patients. *Br. Med. J.*, **2**, 797.

LISTER, T. A., CULLEN, M. H., BREARLEY, R. B. & 7 others (1978) Combination chemotherapy for advanced non-Hodgkin's lymphoma of unfavourable histology. *Cancer Chemother. Pharmacol.*, **1**, 107.

MCAFEE, J. G. & THAKUR, M. L. (1976) Survey of radioactive agents for *in vitro* labelling of phagocytic leukocytes. I. Soluble agents. *J. Nucl. Med.*, **17**, 480.

MACKINTOSH, F. R., O'NEILL, M. & ROSENBERG, S. (1980) Prognostic factors in advanced histiocytic lymphoma. *Proc. Am. Soc. Clin. Oncol.*, **21**, 465.

MILLER, T. P. & JONES, S. E. (1979) Chemotherapy of localised histiocytic lymphoma. *Lancet*, i, 358.

MONFARDINI, S., BANFI, A., BONADONNA, G. & 4 others (1979) Improved five year survival after combined radiotherapy–chemotherapy for Stage I–II non-Hodgkin's lymphoma. *Int. J. Radiat. Oncol. Biol. Phys.*, **6**, 125.

OFFICE OF POPULATION CENSUSES AND SURVEYS (1980) *Cancer Statistics Survival. Series M.B.*1, *No.* 3. London: HMSO.

PANAHON, A., KAUFMAN, J. H., GRASSO, J. A., FRIEDMAN, M. & STUTZMAN L. (1977) A randomised study of radiation therapy versus radiotherapy and chemotherapy in Stage IA–IIIB non-Hodgkin's lymphoma. *Proc. Am. Soc. Clin. Oncol.*, **18**, 321.

RANNIE, G. H. & FORD, W. L. (1977*a*) Physiology of lymphocyte recirculation in animal models. *Proc. IXth Int. Cong.* Lyons.

RANNIE, G. H., THAKUR, M. L. & FORD, W. L. (1977*b*) Experimental comparison of radioactive labels with potential application to lymphocyte migration studies in patients. *Clin. Exp. Immunol.*, **29**, 509.

SCARFFE, J. H., PRUDHOE, J., GARRETT, J. V. & CROWTHER, D. (1980) Colchicine ultrasensitivity of peripheral blood lymphocytes from patients with non-Hodgkin's lymphoma. *Br. J. Cancer*, **41**, 593.

SCHEIN, P. S., CHABNER, B. A., CANELLOS, G. P., YOUNG, R. C., BERARD, C. & DE VITA, V. T. (1975) Potential for prolonged disease free survival following combination chemotherapy of non-Hodgkin's lymphoma. *Blood*, **43**, 181.

SKARIN, A., CANELLOS, G., ROSENTHAL, D., CASE, D., MOLONEY, W. & FREI, E. III (1980) Therapy of diffuse histiocytic and undifferentiated lymphoma with high dose Methotrexate and Citrovorin factor rescue (MTX/CF), Bleomycin, Adriamycin, Cyclophosphamide, Oncovin and Decadron (M-BACOD). *Proc. Am. Soc. Clin. Oncol.*, **21**, 463.

SMITH, M. E., MARTIN, A. F. & FORD, W. L. (1980) Migration of lymphoblasts in the rat. Preferential localisation of DNA-synthesising lymphocytes in particular lymph nodes and other sites. *Monogr. Allergy*, **16**, 203.

SULLIVAN, K., NEIMAN, P., FAREWELL, L. V., HARRISON, D., RUDOLPH, R., EINSTEIN, A. & BAGLEY, C. (1979) Combined modality therapy in advanced diffuse non-Hodgkin's lymphoma. A two and one half year follow up. *Proc. Am. Soc. Clin. Oncol.*, **18**, 442.

THOMSON, A. E. R., O'CONNOR, T. W. E., & WETHERLEY-MEIN, G. (1972) Killing and characterising action of colchicine *in vitro* on lymphocytes of chronic lymphocytic leukaemia. *Scand. J. Haematol.*, **9**, 231.

TIMOTHY, A. R., LISTER, T. A., KATZ, D. & JONES, A. E. (1979) Localised non-Hodgkin's lymphoma. *Eur. J. Cancer*, **16**, 799.

WAGSTAFF, J., GIBSON, C., THATCHER, N. & 4 others (1981*a*) A method for following human lymphocyte traffic using Indium-111 oxine labelling. *Clin. Exp. Immunol.*, **43**, 435.

WAGSTAFF, J., GIBSON, C., THATCHER, N., FORD, W. L., SHARMA, H. & CROWTHER, D. (1981*b*) Human lymphocyte traffic assessed by Indium-111 oxine labelling—clinical observations. *Clin. Exp. Immunol.*, **43**, 443.

WARNKE, R., MILLER, R., GROGAN, T., PEDERSEN, M., DILLEY, J. & LEVY, R. (1980) Immunologic phenotype in 30 patients with diffuse large cell lymphoma. *N. Engl. J. Med.*, **303**, 293.

YOUNG, R. C., HOWSER, D. M., ANDERSON, T., JAFFE, E. & DE VITA, V. T. (1979) CNS infiltration: A complication of diffuse lymphomas. In *CNS Complications of Malignant Disease.* Eds Whitehouse & Kay. London: Macmillan Press. p. 121.